# Supplementary material for: Reuse of intermittent catheters: a qualitative study of IC users’ perspectives
Source: BMJ Open. 2018 Aug 17;8(8):e021554. doi: 10.1136/bmjopen-2018-021554 (PMC6104744; doi:10.1136/bmjopen-2018-021554)
Supplement: Supplementary file 1 [file bmjopen-2018-021554supp001.pdf]

MULTICATH MODULE 2 SCHEDULE FOR ITERATION 3

Background questions

1. Experience of intermittent catheterisation:

    How long have you been using intermittent catheterisation?

    Do you self-catheterise? Or does somebody help you?

    How many times per day do you catheterise?

    What catheter do you use?

    Do you catheterise:

        At home only?

        When you are out?

        If yes, when you are out what kind of catheter do you use, is it the same one as for home use?

Beliefs and practices re IC routine

2. Beliefs and concerns about IC rituals, catheters, infection and sterility

    What steps do you take before you do IC?

    What is the reason you take these steps? (Probe without leading for whether or not they value sterility)

    Is your IC routine clean or sterile?

    What is sterile to you? (prompt – how is it different to clean?) What do you mean by clean?

Experience of urine infection

3. During your time using intermittent catheterisation have you ever experienced a urine infection?

    If yes, how often would you say you have a urine infection?

    Can you recall the last time you had a UTI? What happened?

4. What it feels like to have a urine infection:

    Can you describe how you feel when you think you may be developing a urine infection?

    Does anything happen beforehand when you think one is coming on?

How do you think your infection came about? Prompt (Where do you think the germs come from when you get a UTI?)

What do you do to prevent an infection?

What advice do you get/have you had from your health care professional to prevent infections?

Complete the table – UTI signs and symptoms: Where patients name a symptom or sign check with them the terminology and that the patient is happy that it sits within a category on the table. We want to understand the terminology that patients use to describe UTI symptoms.

Looking at the symptoms listed here, are these the right things that describe a urine infection from your experience? Is anything missing?

5. Seeking help:

When you feel you may have a urine infection, what do you do about it?

Prompt: antibiotics

At what point do you seek help from others?

Who do you seek help from? And what do you do?

Cleaning questions

6. Methods of cleaning:

Have you ever re-used your catheter?

If Yes, under what circumstances? Did you clean it after its first use? How did you clean it? How did you store it between uses?

What is/would be acceptable to you?

If No, Have you ever thought about re-using your catheters?

If you were offered a method of cleaning and re-using your catheters instead of using a new one each time, what kinds of things would be important to you? [Prompts: How to clean; Where to clean; Where to store between uses]

What kind of cleaning regime is acceptable to you? [Prompts: what cleaning methods and products, what about storage storage/travelling, and lubrication?]

What would worry you about re-using your catheter? [Prompts: cleaning in a public loo, being at work, storing and transporting]

What would be good about re-using?

Discrete choice experiment questions

[I would now like to discuss with you the benefits and drawbacks of using disposable and reusable

Catheters]

7. What is good about your current catheter?

What do you think about single-use catheters?

What are the benefits of using a disposable or single-use catheter?

What are the drawbacks?

[in the following situations]

- At home?
- At work?
- On holiday?
- In a public place?
- Going out?
- Other?

What do you think about the idea of a re-usable catheter?

- What do you see as a benefit of re-using your catheter?
- What do you see as a drawback in re-using your catheter?

[in the following situations]

- At home?
- At work?
- On holiday?
- In a public place?
- Going out?
- Other?

- What would encourage you to use it?
- What would discourage you from using it?

Preamble: We know from previous interviews/speaking to other patients, that avoidance of infection is what matters most to everyone. We also know that other things are highly important such as:

- a comfortable insertion,
- having a catheter that is easy to use and handle,
- that comes in packaging which is easy to open,
- that your catheter is discrete,
- that you can trust your catheter/ trust in the product

As we already know all of this, we now want to explore other things around single-use catheters and re-usable catheters.

Together let’s go through each item in this list and tell me what you think about each one? Your comments are very valuable to us.

Those relating to disposable catheters:

1. That I don’t have the bother of cleaning the catheter
2. That I don’t need to have special equipment for cleaning the catheter
3. That I don’t have to carry used/soiled catheters around with me in my bag

Those relating to re-using catheters:

4. That I have less catheters to carry with me on holiday
5. That my catheters don’t cost the NHS more than they need to/too much
6. That I don’t have to store lots of catheters at home
7. That I don’t have to worry that I will run out of catheters
8. That I don’t have to get rid of lots of rubbish when I am out
9. That my catheters are not bad for the environment.
