# Supplementary material for: Reuse of intermittent catheters: a qualitative study of IC users’ perspectives
Source: BMJ Open. 2018 Aug 17;8(8):e021554. doi: 10.1136/bmjopen-2018-021554 (PMC6104744; doi:10.1136/bmjopen-2018-021554)
Supplement: Author's manuscript [file bmjopen-2018-021554.draft_revisions.pdf]

BMJ Open is committed to open peer review. As part of this commitment we make the peer review history of every article we publish publicly available.

When an article is published we post the peer reviewers' comments and the authors' responses online. We also post the versions of the paper that were used during peer review. These are the versions that the peer review comments apply to.

The versions of the paper that follow are the versions that were submitted during the peer review process. They are not the versions of record or the final published versions. They should not be cited or distributed as the published version of this manuscript.

BMJ Open is an open access journal and the full, final, typeset and author-corrected version of record of the manuscript is available on our site with no access controls, subscription charges or pay-per-view fees (http://bmjopen.bmj.com).

If you have any questions on BMJ Open's open peer review process please email info.bmjopen@bmj.com

BMJ Open

Re-use of intermittent catheters: A qualitative study of IC users' perspectives

Journal:	*BMJ Open*
Manuscript ID	bmjopen-2018-021554
Article Type:	Research
Date Submitted by the Author:	10-Jan-2018
Complete List of Authors:	Avery, Miriam; University of Southampton, Faculty of Health Sciences<br>Prieto, Jacqui; University of Southampton, Faculty of Health Sciences<br>Okamoto, Ikumi; University of Southampton, Faculty of Health Sciences<br>Cullen, Samantha; University of Southampton, Faculty of Health Sciences<br>Clancy, Bridget; University of Southampton, Faculty of Health Sciences<br>Moore, Katherine ; University of Alberta<br>Macaulay, Margaret; University of Southampton, Faculty of Health Sciences<br>Fader, Mandy; University of Southampton, Faculty of Health Science
Keywords:	Intermittent catheterisation, re-use, single-use, qualitative interviews

SCHOLARONE™
Manuscripts

For peer review only

TITLE PAGE

Title of the article

Re-use of intermittent catheters: A qualitative study of IC users' perspectives

Corresponding author
Miriam Avery
Address: Faculty of Health Sciences, University of Southampton, Southampton, UK
Tel.: 02381 204141
Email: M.Avery@soton.ac.uk

Co-authors

- Jacqui Prieto - Faculty of Health Sciences, University of Southampton, University Road, Southampton, SO17 1BJ, UK.
- Ikumi Okamoto – Faculty of Health Sciences, University of Southampton, University Road, Southampton, SO17 1BJ, UK.
- Samantha Cullen – Faculty of Health Sciences, University of Southampton, University Road, Southampton, SO17 1BJ, UK.
- Bridget Clancy - Faculty of Health Sciences, University of Southampton, University Road, Southampton, SO17 1BJ, UK.
- Katherine. N. Moore - Faculty of Nursing, University of Alberta, Edmonton, Canada.
- Margaret Macaulay - Faculty of Health Sciences, University of Southampton, University Road, Southampton, SO17 1BJ, UK.
- Mandy Fader - Faculty of Health Sciences, University of Southampton, University Road, Southampton, SO17 1BJ, UK.

Word count (excluding title page, abstract, references, figures and tables) = 3941

Funding

This paper refers to independent research funded by the National Institute for Health Research (NIHR) under its Programme Grants for Applied Research (PGfAR) (Grant Reference Number RP-PG-0610-10078).

For peer review only

ABSTRACT

Objectives

To explore the views of intermittent catheter (IC) users regarding the potential for re-using uncoated catheters.

Design

Qualitative study with semi-structured interviews. The interviews were recorded, transcribed and analyzed thematically.

Setting

Participant's own homes in Hampshire and Dorset, UK.

Participants

A convenience sample of 39 IC users, aged 23-86 years, using IC for at least 3 months.

Results

For many participants, the main concern regarding re-use of IC catheters was safety, with the fear that re-use could increase the risk of urinary tract infection (UTI). The practicalities of cleaning methods (extra products, time and storage) were also considered to be potentially burdensome. Some participants were concerned about the environmental impact (waste) and cost of single-use catheters and considered this an advantage of re-use. The fear of running out of catheters was also identified as a reason why re-use may be advantageous. One participant felt that being able to re-use their catheter would mean 'you'd know you've always got one.' The potential for re-use of catheters was also dependent on location and availability of public toilets. The interview findings revealed that often the disadvantages of single-use could be off-set by the advantages of re-use and vice versa; for example, the need to take many single use catheters on holiday could be addressed by re-use, whilst the burden of cleaning would be obviated by single-use.

Conclusions

If shown to be safe with a practical cleaning method, some participants would find re-use an acceptable option, alongside their current single-use method. The choice to use a mixture of single-use and re-using catheters for different activities (at home, work or holiday) could optimise the advantages and disadvantages of single-use and re-use. The safety and acceptability of such an approach would require testing in a clinical trial.

Strengths and limitations of the study

- We believe this is the first study to explore IC users' perspectives on the advantages and disadvantages of single-use and re-use of catheters.
- Re-use is rare in the UK and the perspectives of single-use IC users about re-using catheters were therefore hypothetical.
- The researcher was part of a team investigating potential re-use of catheters and might have elicited socially desirable responses from interviewees; however, she presented as an impartial observer during the data collection.

For peer review only - http://bmjopen.bmj.com/site/about/guidelines.xhtml

INTRODUCTION

Intermittent catheterisation (IC) is well established management for children and adults with chronic urinary retention. The positive benefits of IC are well-known; IC users have reported relief of symptoms of frequency, urgency and incontinence, improved sleep and fewer restrictions on daily physical activities,(1, 2) and improved quality of life (QOL).(3) Although the value of IC is clear, some users describe the challenges they encounter. They report negative effects on QOL, both psychologically and socially, and urinary tract infection (UTI) remains a common problem.(1, 2) In one cross-sectional survey study only half of the 44 participants were completely satisfied with IC.(4)

Research about IC-related issues, in particular UTI, has focussed on single-use catheters (the catheter is used once then discarded) with the development of new catheter coatings, pre-lubrication and compact designs. Re-use of uncoated catheters (the same catheter cleaned and re-used by one individual several times) has gained little attention from researchers or industry, even though single-use catheters may not be the most environmentally friendly or cost effective strategy. Catheter re-use is common in some affluent countries (e.g. Australia, Japan and Canada), but is now rare in the UK since the introduction of single-use coated catheters and subsequent changes in medical device legislation. An International guideline, prepared by the Infectious Diseases Society of America (IDSA)(5) concluded that for those living in the community and using clean self-catheterisation, the differences between sterile catheters (single-use) or reused catheters are not significant and that there is no convincing evidence that one catheter design or method is better than another in terms of UTI. This leaves clinicians with insufficient information to recommend one approach over the other.

A review of the literature was conducted and four papers were found which, to varying degrees, included user experiences of re-using IC catheters compared to single-use.(6-9) Two were qualitative studies exploring individuals' experiences of IC in general; issues raised included cost and the environmental impact of single-use compared to re-use, from a UK and US perspective.(6, 7) In two other studies,(8, 9) children with spina bifida and their families were asked about experiences of using a new hydrophilic coated single-use catheter, compared to their usual method of re-using an uncoated catheter. Some described the single-use catheter as more hygienic and felt that it was 'safer' for their child.(9) Single-use was also rated positively regarding convenience and for outings where the toilet facilities were not satisfactory.(8, 9) However, the packaging and the environmental impact of single-use catheters was raised by parents as a downside of single-use catheters.(9)

Although the potential benefits of re-use may appeal to some IC users, the potential risks and challenges are not well understood. This study is part of the National Institute for Health Research (NIHR) funded Mult*I*Cath programme (10) which aims to develop and test a catheter cleaning method utilising laboratory techniques and patient panels as well as utilising interviews and surveys to determine IC user and clinician perspectives (11) regarding re-use of catheters.

For peer review only - http://bmjopen.bmj.com/site/about/guidelines.xhtml

The purpose of this study is to explore the views of community dwelling IC users regarding the advantages and disadvantages of single-use or re-use of uncoated catheters.

METHOD

This study used a qualitative in-depth interview method. Findings from a subset of these interviews, related to the views of IC users about UTI symptoms and management, has been recently published.(12) A convenience sample of 39 IC users was recruited from 12 GP practices in Hampshire and Dorset, UK. Potential participants were identified via a computerised database search using the prescription code. GP checked eligibility (see Box 1) and an invitation letter with a reply form, Participant Information Sheet and FREEPOST envelope were sent out by practice staff. At three weeks, reminders were sent to non-responders. Recruitment for the study continued until it was deemed data saturation had been reached. Ethics permission was granted from an NHS Research Ethics Committee (REC reference 13/LO/1511). Informed written consent was obtained from each participant and all information was kept confidential.

Box 1: Inclusion and exclusion criteria

Inclusion criteria:

- Over 18 years
- Currently using intermittent catheters for three months or more

Exclusion criteria:

- Urethral stricture or deformity
- Immune deficiency disorder (increased infection risk)
- External carer required for IC (e.g. visiting community nurse performs IC)

Data collection

All interviews were conducted face-to-face in participants' own homes by one of three trained female interviewers, including a qualitative researcher (SS) and two research nurses (BC and MA). None of the interviewers had been in previous contact with any of the participants and they attended in a non-clinical capacity as non-judgemental listeners. A relative or carer was present for the interview if requested by the participant. A semi-structured interview schedule was used to explore experiences, values and beliefs about IC (see Appendix 1: interview schedule (Supplementary file 1)). Each interview lasted approximately 45-60 minutes. Interviews were recorded using digital audio recording equipment and then transcribed verbatim. All transcripts were anonymised with an assigned study number. The transcripts were read only by the researchers and not by participants. Field notes were made during interviews, which were used in the analysis.

For peer review only - http://bmjopen.bmj.com/site/about/guidelines.xhtml

Data analysis

Data from interviews and field notes were analysed thematically and coded using NVivo 10 (QSR International) by a qualitative researcher (IO) who had not conducted the interviews. This involved moving back and forth between interview transcripts and field notes, with reference to the research literature. An initial coding framework was derived from the first 20 interview transcripts. This was further refined through discussion with the wider research team and ongoing analysis of the remaining transcripts. Any newly emerged themes that did not fit into the initial themes were discussed as a team. No participants were involved in the analysis of data.

RESULTS

Participants

A total of 139 IC users were invited to take part. Seventy-four (53%) responded, of whom 39 were willing to participate and eligible. The participants included 24 men and 15 women, with a mean age of 67 years (range 23-86 years) who had been using IC for a mean length of 8.5 years (range 9 months – 30 years). The frequency of IC ranged from once every second day to 10 catheterisations a day (mean 4/day). Reasons for IC included chronic urinary retention (n=20), neurological impairment (n=10) and other factors such as post-surgery or post-chemotherapy (n=9).

The majority of participants (n=34) were currently using a catheter once and discarding it after use. Most of these participants were using hydrophilic coated catheters and 10 used catheters with a compact design. Of the remaining five, four were currently reusing catheters on an on-going basis, and one had previously re-used catheters, switching to single-use 10 years ago. Table 1 shows the characteristics of the participants with experience of re-using catheters, all of whom were men.

Table 1: Characteristics of participants with experience of re-using catheters (TABLE REMOVED TO PROTECT PARTICPANTS IDENTITIES)

Qualitative findings

The results focus on the perspectives of the participants regarding single-use and re-use of IC catheters. Themes emerged about safety, burden and lifestyle. Those who had never re-used catheters were asked about their views but not their own experiences.

Overall, around a third of the single-use participants indicated they would not want to re-use IC catheters and would not find it an acceptable method. For the remaining participants, the potential acceptability of re-use was very much dependent on the safety and efficacy of the cleaning regimen and whether there was an increased risk of infection, as well as factors such as convenience and practicalities (Figure 1).

For peer review only

Uploaded image: *Figure 1: Factors affecting acceptability of re-use*

1. Concerns regarding risk of UTI

1.1 Safety: Fears about increasing risk of UTI

The question of UTI risk was the main concern raised by many single-users when asked about re-using catheters.

> *Infection [concerns me about re-using catheters] definitely. Especially as I've not had any infection, I think I'm doing something right, if you get me. (Participant 22, female, single-user)*

> *My honest opinion [about reusing] is I can understand why they're trying to do it, it's they're trying to save money. But in my opinion there's always a chance that that's going to go wrong and I'm the one who's gonna end up having a water infection or something like that. (Participant 13, male, single-user)*

Most perceived that using single-use catheters meant they had less chance of getting an infection.

> *[Single-use catheters have] less chance on infection, you can dispose of it, get rid of it. (Participant 40, male, single-user)*

However, the participants who were currently re-using were not deterred by fears about infection.

1.2 Necessity of safe cleaning method

Related to the fears described above, some single-users described the importance of an effective catheter cleaning method as a pre-requisite for re-use.

> *[It is important] that you follow all the steps that you are supposed to. Because if you don't then it might not be sterile. Because I don't really know what it, what those effective ways would be. (Participant 16, female, single-user)*

For peer review only - http://bmjopen.bmj.com/site/about/guidelines.xhtml

2 Cleaning, preparation, and storage

2.1 Concerns about a complicated/time-consuming cleaning procedure

Most single-users thought that the cleaning procedure would be complicated and were concerned about the amount of work and time involved.

> *How much labour would be to get it back to the required state of sterility that I needed? And it was clean, clean to be reusable. To me, those in my mind are quite important matters which I would find. (Participant 12, male, single-user)*

> *[Time] is the only thing. If there's too much preparation time then I wouldn't be interested […]. (Participant 10, male, single-user)*

Some single-users mentioned that they needed to know more about the cleaning method before they could comment on re-use.

It appeared, however, that all the current catheter re-users were conducting a very simple catheter cleaning procedure, typically using only tap water to rinse out used catheters.

> *Holding it under the tap [running it under tap water], and then I put it right back in the pouch, and then it's as clean as a whistle. […] (Participant 19, male, re-user)*

2.2 Concerns about storage and supply of catheters

The participants who would consider re-use of catheters thought it could save space in the home. However, some suggested that finding space to store extra equipment for cleaning catheters would be a problem. One participant, with a complex long-term health condition that required other equipment, was concerned about this due to the amount of equipment she already had.

> *My bathroom is already clogged full of all my bulky stuff, I already have my sort of like bag that hangs up for flushing my bowels out.[…] I think that I'd want it to not be too bulky. (Participant 11, female, single-user)*

In terms of catheter supply, although some participants were very happy with their current supply arrangements, for others the prospect of a supply shortage was a drawback of single-use catheters. Some participants thought they would not need to worry about running out if they could re-use them.

For peer review only - http://bmjopen.bmj.com/site/about/guidelines.xhtml

Wouldn't have to keep running up the chemist [if I used reusable catheters]. You'd know you've always got one. You would know there's always one there. (Participant 7, female, single-user)

3 Social responsibility

3.1 Reducing cost on NHS

Many participants expressed their concerns about the financial cost of their catheters on the National Health Service (NHS) and some suggested that they would consider re-use to contribute to reducing such cost.

I think the GP's surgery pays for [my catheter] but I found out how much it was last week and I was shocked, really shocked actually. I feel guilty about that. (Participant 15, female, single-user)

I would [be happy to reuse], because I was once told years ago that I'm one of their most expensive patients. I would like to try and cut down the cost I am to them. (Participant 5, female, single-user)

3.2 Reducing waste and the impact on the environment

Several participants felt that re-using catheters would reduce waste and, for that reason, might be more environmentally friendly.

The good thing about re-using would be that it would cut down the amount of waste. If I go through [single-use catheters] every 6 weeks that is a mountain of catheters every year I get though. So I think that is the main benefit really. (Participant 38, female, single-user)

A participant who was currently re-using their catheter indicated that not disposing of the catheter after each use alleviated the worry about contributing to landfill waste. Another described how reducing waste was the main motive to start reusing their catheters.

I hate wasting anything, and so that's all built into why I use the catheter more than once. (Participant 25, male, re-user)

For peer review only - http://bmjopen.bmj.com/site/about/guidelines.xhtml

4 Practicalities and Location

4.1 Freedom and a normal life

Most single-users appreciated their current catheters as they were convenient, comfortable and easy to use, with some describing them as giving freedom and a normal life.

> *I love that [my catheter] is compact. They are just so convenient to use. No fuss. It doesn't impact on your normal daily lifestyle. They are great for travelling. That's what I love about my catheters. And it gives me a normal life; otherwise I would probably have to be at home more really. (Participant 38, female, 48)*

When considering re-use, the participants discussed the locations where they would be more likely to re-use their catheters; they often compared the practicalities of re-use inside and outside the home as well as issues around travel.

4.2 Travel

The compact size of single-use catheters was appreciated by participants because they were easy to carry individually. However, for holidays, the need to take a large supply was a hindrance. In this situation, participants noted that re-use of catheters could be a more practical option.

One current re-user valued this aspect of reusing from his experience.

> *If I take [my catheters], I'll take 10 for the month or something. If I take the other one I've got to take 120. […] I did a trip around Australia once with my family and we were away 30 days or a month and the difference would have been huge. I pretty much would have needed a suitcase just for that. So that's always been a major issue. (Participant 37, male, re-user)*

However, many single-users were concerned about carrying the cleaning equipment on holiday.

> *If you go on holiday for a week, how many bottles would you have to take? And you wouldn't get through customs, or you wouldn't get through, because you'd have all these fluids. (Participant 22, female, single-user)*

For peer review only - http://bmjopen.bmj.com/site/about/guidelines.xhtml

4.3 Carrying used catheters

Many participants appreciated the portability and ease of disposal of the single use catheters. Some single-users, therefore, wondered what they would have to do with the used catheters if they catheterised when they were out.

> *If you are going out for the day. You'd use your catheter in the morning; I don't want to take that one around with me. I don't know how I could. (Participant 41, male, single-user)*

However, one of the re-users emphasised the possible flexibility of reusing their catheter, as they could also be thrown away after a single use if needed.

> *You can always throw it away and you've only done the same as you've done with a non-re-usable one. So if you find yourself out and about and it's not as hygienic as you'd have liked; [...] I will tend to use it once and throw it away under those circumstances. (Participant 37, male, 50, single-user)*

4.4 Discretion

The majority of single-users appreciated the compact packaging and discreetness of their current catheters. Many were concerned they would not have these advantages if they re-used catheters and the lack of discreetness would restrict them.

> *[Single-use catheters are] just quick really, it doesn't take me any longer than somebody else going into the toilet, not much longer at all so it's that easy. If you have to start cleaning it after using it….while you're out, it then gets a bit [much]. And it's obviously difficult to do it in a public toilet as well. (Participant 3, female, single-user)*

While some said that they would be happy to re-use at home, others expressed their concern, as they did not want visitors to see used catheters or the cleaning equipment.

> *How would you clean [the catheter]? If you've got a container, do you leave it in the toilet? I just think if you've got friends coming round, I don't want them seeing that. (Participant 41, male, single-user)*

4.5 Choice of catheters – mixed use

Among those who viewed re-use as potentially acceptable, most said they would value having both types of catheters to choose from depending on the situation.

For peer review only - http://bmjopen.bmj.com/site/about/guidelines.xhtml

Important stuff would be that when I'm doing it at home it's not generally a problem. If I know what I've got to do I will do it. It's knowing that when you are going away, you don't know where you are going to, […} is that equipment there? For me to be able to do it? (Participant 1, male, single-user)

…..I wouldn't want to be forced to have just re-used catheters. But in most circumstances I would choose to use it accept for when I'm travelling in which case I would definitely want to be using the disposable ones. And I think generally speaking if you are away you don't want all the faff. (Participant 38, female, single-user)

An overall summary of the perceived advantages and disadvantages of single-use and re-use of catheters from the participants in this study is found in Table 2.

Table 2. Summary of the perceived advantages and disadvantages of single use and re-use

**Re-use: Advantages**	**Re-use: Disadvantages**
• Positive impact on environment – less waste<br>• Cost saving<br>• Fewer catheters to carry when travelling<br>• Always having a catheter available<br>• Fewer catheters to store	• Concern about hygiene and perceived link to UTI<br>• Burden of cleaning and preparation<br>• Storing cleaning equipment<br>• The need to take cleaning equipment on holiday<br>• The need to carry used catheters<br>• Lack of discreetness associated with the cleaning method
**Single-use: Advantages**	**Single-use: Disadvantages**
• Sterile and therefore considered less likely to cause infection<br>• Instantly useable<br>• Easy to use/dispose<br>• Easy to carry and discreteness<br>• Comfortable to use/pain free<br>• Good delivery service<br>• Gives freedom and normality	• Negative impact on environment – more waste<br>• Higher cost<br>• Large quantities of catheters to carry on holiday<br>• Potential shortages of supply and failure of delivery service<br>• Difficult to dispose in public toilets

DISCUSSION

We believe this is the first study to explore IC users' perspectives on the advantages and disadvantages of single-use and re-use of catheters. The findings highlight the complexity of factors involved and show the variation of views amongst IC users; for some, re-use is not an acceptable option and for others it could be acceptable, but robust evidence is needed regarding safety, and the cleaning method must be shown to be effective and user-friendly.

Safety

For peer review only - http://bmjopen.bmj.com/site/about/guidelines.xhtml

For IC users to consider re-use they must first be satisfied that it is safe to do so; this was the crucial factor for many. In fact, around a third of participants in this study would not currently consider re-use mainly due to fears about infection. A guideline for the diagnosis, treatment and prevention of catheter associated UTI (IDSA) concluded that there have not been enough robust studies to provide evidence regarding risk of UTI when comparing re-use of catheters to single-use catheters.(5) This emphasises the need for a randomised controlled trial to properly examine this.

Patient-centred cleaning method

The development of a cleaning method which is practical for IC users was an issue raised by participants. Concerns were voiced about the number of cleaning items requiring storage and the potential burden of doing the cleaning as well as the effectiveness of the cleaning method. Others have suggested that the cleaning method could be a burden when re-using catheters (13) which could in turn impair independence and compliance;(14) Chick et al (2015) found that some families in their study preferred the convenience of the one-step process for single-use catheters.(8) It is therefore important that the cleaning method is developed with IC users alongside rigorous laboratory testing.(15)

Social responsibility

Despite participants' concerns about re-use, they recognised some advantages. Many thought there could be cost savings for the NHS and a positive environmental impact and some were currently re-using, or would consider re-using, for these reasons. Similar findings were reported by Kelly et al in which 16 individuals were interviewed about their experiences and concerns about IC use. Financial burden to the health service and environmental impact were raised by 63% (10/16) and 38% (6/16) of the participants respectively; the authors suggested this reflects the greater awareness of environmental issues in society.(6) Although several of the participants in this study felt that re-use of catheters would be more environmentally friendly, this is a complex issue dependent on the method used for cleaning; there is a need for knowledge to be gained on environmental issues.

Four distinct modelling studies on cost effectiveness using different IC catheters and methods have been published. Both Neovius(16) and Bermingham(17) compared re-use with single-use catheters. In a conference abstract, Neovius concluded that re-use of PVC catheters was not cost effective compared to single-use coated catheters,(16) whereas Bermingham indicated that re-use could be more cost effective than single-use catheters but that additional evidence was required before any practice changes could be recommended(17). Clark and colleagues(18) concluded that single-use coated rather than single-use uncoated catheters were more cost effective when outcomes were calculated over a patient's lifetime. And finally, Neovius and Lundquist(19) indicated that patients would be willing to pay out of pocket for a hypothetical catheter that would reduce/prevent UTIs. The probabilistic decision model of these studies regarding risk of UTI and compliance limits the interpretation of the results and indicates, as Bermingham stated, that further robust evidence is required.

For peer review only - http://bmjopen.bmj.com/site/about/guidelines.xhtml

Time and place

Although many of the participants would consider catheter re-use, willingness to do so varied between individuals based on lifestyle and circumstances. The need for discretion was of great concern and was closely linked to location; some participants would only consider re-using catheters when they were at home. The literature also confirms that location, and specifically whether at home or away from home, has a bearing on how people feel about re-use.(8, 9) This highlights the need for a flexible approach to IC methods, which may be achieved by giving IC users the option to use both catheter types to suit their lifestyle and preferences.

Many of the perceived disadvantages of re-use are off-set by advantages of single-use and vice versa (see Table 2). For example, some IC users were concerned about running out of their single use catheters, and the ability to re-use their catheter would mean they always have a usable catheter. Others emphasised the benefit of using single-use catheters when in public places and the difficulties of re-use when away from home reinforcing the potential benefit of mixed use. Furthermore, if re-use of catheters was more commonplace, efforts could be made to design and manufacture discreet, re-usable versions in the same way as for single-use.

Limitations

As the primary aim of the study was to explore whether reusing catheters would be acceptable to IC users in preparation for a randomised controlled trial, and this was stated in the participant information sheet, we may have recruited primarily individuals who were interested in issues around re-use. Therefore, findings may not be representative of all UK IC users. Furthermore, the perspectives of single-use IC users about re-using catheters were hypothetical and not based on their actual experiences. Also the researcher's presence during the data collection might have elicited socially desirable responses; however, the researcher presented as an impartial observer during the data collection.

Implications for future research/clinicians/policy makers

This study has shown that some IC users would find re-use of catheters acceptable, but there is a need to develop a rigorous cleaning method for IC catheters which is effective and user-friendly. If shown to be safe, then efforts could be made with the design to develop user friendly versions with an efficient cleaning method.

CONCLUSION

This study revealed that if shown to be safe with a practical cleaning method, some participants would find re-use of catheters for some of the time an acceptable option. The choice to use a mixture of single-use and re-use of catheters for different activities (at home, work or holiday) could optimise the advantages and disadvantages of both methods. With environmental issues and cost high on the public agenda, some IC users would advocate that if re-use is shown to be

For peer review only - http://bmjopen.bmj.com/site/about/guidelines.xhtml

safe and acceptable then it could be made available as an option. The safety and acceptability of such an approach would require testing in a clinical trial.

Acknowledgements
We would like to thank all the interviewees who agreed to take part and share their experiences.

Contributors
MF was the principal investigator and was primarily responsible for the original grant application. MF, JP, MM, SC and MA were involved in designing the study and methods. JP led the overall study team. SC collected the majority of the data. BC and MA contributed to the data collection. IO led the analysis and IO and MA were involved in interpretation of the qualitative data and wrote draft manuscripts. IO, JP, MA, KNM, MM and MF wrote drafts and/or critically revised the manuscripts.

Patient consent
Detail has been removed from this case description/these case descriptions to ensure anonymity.

Competing interests
None declared

Data sharing statement
No additional data is available.

REFERENCE LIST

1. McConville A. Patients' experiences of clean intermittent catheterisation. *NTplus* 2002;98(4):55-6.
2. Shaw C, Logan K, Webber I, et al. Effect of clean intermittent self-catheterization on quality of life: a qualitative study. *J Adv Nurs* 2008;61(6):641-50.
3. Kessler TM, Ryu G, Burkhard, FC. Clean Intermittent self-Catheterization: a burden for the patient? *Neurourol Urodyn* 2009;28:18-21.
4. Bolinger R, Engberg S. Barriers, complications, adherence, and self-reported quality of life for people using clean intermittent catheterization. *J Wound Ostomy Continence Nurs* 2013;40(1):83-9.
5. Hooton TM, Bradley SF; Cardenas, DD, et al. Diagnosis, prevention, and treatment of catheter-associated urinary tract infection in adults: 2009 international clinical practice guidelines from the Infectious Diseases Society of America. *Clin Infect Dis* 2010;50:625-63.
6. Kelly L, Spencer S, Barrett G. Using intermittent self-catheters: experiences of people with neurological damage to their spinal cord. *Disabil Rehabil* 2014;36(3):220-6.
7. Wilde MH, Brasch J, Zhang Y. A qualitative descriptive study of self-management issues in people with long-term intermittent urinary catheters. *J Adv Nurs* 2011;67(6):1254-63.

For peer review only - http://bmjopen.bmj.com/site/about/guidelines.xhtml

8. Chick HE, Hunter KF, Moore KN. Parent and child experiences using a hydrophilic or reused PVC catheter for intermittent catheterisation. *J Clin Nurs* 2012;22:513-20.
9. Kiddoo D, Sawatzky B, Bascu C-D, et al. Randomized crossover trial of single use hydrophilic coated versus multiple use polyvinylchloride catheters for intermittent catheterization to determine incidence of urinary infection. *J Urol* 2015;194:174-79.
10. Fader M. Development and clinical trial of a mixed (multi/single-use) catheter management package for users of intermittent catheterisation. *National Institute for Health Research (NIHR) Programme Grants for Applied Research (PGfAR)*. Programme (Grant Reference Number RP-PG-0610-10078), 2013
11. McClurg D, Coyle J, Long A, et al. A two phased study on health care professionals' perceptions of single or multi-use of intermittent catheters. *Int J Nurs Stud* 2017;72:83-90.
12. Okamoto I, Prieto J, Avery M, et al. Intermittent catheter users' symptom identification, description and management of urinary tract infection: a qualitative study. *BMJ Open* 2017;7: e016453. doi:10.1136/bmjopen-2017-016453.
13. Woodward S. Improving quality of life for men using intermittent self-catheterisation. *British Journal of Neuroscience Nursing* 2013;9(3):114-9.
14. Dean GE. Are single use catheters worth the expense? *J Urol* 2015;194(1):12-13.
15. Wilks S, Morris N, Delgado D, et al. Development of an effective and acceptable cleaning method to allow safe re-use of plain, uncoated catheters for intermittent catheterization. *Neurourol Urodyn* (Abstract 566) 2016;4:449-50.
16. Neovius K, Hakansson M, Lundqvist T. Cost consequences of single-use and re-use of urinary catheters among patients performing daily intermittent catheterisation (Abstract PMD43). *Value in Health* 2014;18:A351.
17. Bermingham SL, Hodgkinson S, Wright S, et al. Intermittent self catheterisation with hydrophilic, gel reservoir, and non-coated catheters: a systematic review and cost effectiveness analysis. *BMJ* 2013; 346:e8639
18. Clark JF, Mealing SJ, Scott DA, et al. A cost-effectiveness analysis of long-term intermittent catheterisation with hydrophilic and uncoated catheters.*Spinal Cord* 2016;54:73-77.
19. Neovius KE, Lundqvist T. CIC users' preference for catheters reducing the UTI frequency (Abstract PUK14). *Value in Health* 2014;17:A468.

Figure Legend

Figure 1: Factors affecting acceptability of re-use

For peer review only - http://bmjopen.bmj.com/site/about/guidelines.xhtml

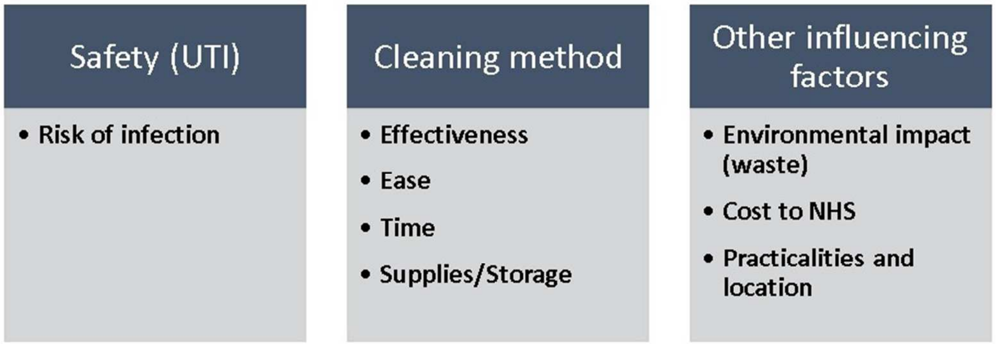

Figure 1: Factors affecting acceptability of re-use

77x27mm (300 x 300 DPI)

MultiCath Module 2 Schedule for iteration 3

Background questions

1. Experience of intermittent catheterisation:

    How long have you been using intermittent catheterisation?

    Do you self-catheterise? Or does somebody help you?

    How many times per day do you catheterise?

    What catheter do you use?

    Do you catheterise:

        At home only?

        When you are out?

        If yes, when you are out what kind of catheter do you use, is it the same one as for home use?

Beliefs and practices re IC routine

2. Beliefs and concerns about IC rituals, catheters, infection and sterility

    What steps do you take before you do IC?

    What is the reason you take these steps? (Probe without leading for whether or not they value sterility)

    Is your IC routine clean or sterile?

    What is sterile to you? (prompt – how is it different to clean?) What do you mean by clean?

Experience of urine infection

3. During your time using intermittent catheterisation have you ever experienced a urine infection?

    If yes, how often would you say you have a urine infection?

    Can you recall the last time you had a UTI? What happened?

4. What it feels like to have a urine infection:

    Can you describe how you feel when you think you may be developing a urine infection?

    Does anything happen beforehand when you think one is coming on?

For peer review only - http://bmjopen.bmj.com/site/about/guidelines.xhtml

How do you think your infection came about? Prompt (Where do you think the germs come from when you get a UTI?)

What do you do to prevent an infection?

What advice do you get/have you had from your health care professional to prevent infections?

Complete the table – UTI signs and symptoms: Where patients name a symptom or sign check with them the terminology and that the patient is happy that it sits within a category on the table. We want to understand the terminology that patients use to describe UTI symptoms.

Looking at the symptoms listed here, are these the right things that describe a urine infection from your experience? Is anything missing?

5. Seeking help:

When you feel you may have a urine infection, what do you do about it?

Prompt: antibiotics

At what point do you seek help from others?

Who do you seek help from? And what do you do?

Cleaning questions

6. Methods of cleaning:

Have you ever re-used your catheter?

If Yes, under what circumstances? Did you clean it after its first use? How did you clean it? How did you store it between uses?

What is/would be acceptable to you?

If No, Have you ever thought about re-using your catheters?

If you were offered a method of cleaning and re-using your catheters instead of using a new one each time, what kinds of things would be important to you? [Prompts: How to clean; Where to clean; Where to store between uses]

What kind of cleaning regime is acceptable to you? [Prompts: what cleaning methods and products, what about storage storage/travelling, and lubrication?]

What would worry you about re-using your catheter? [Prompts: cleaning in a public loo, being at work, storing and transporting]

What would be good about re-using?

For peer review only - http://bmjopen.bmj.com/site/about/guidelines.xhtml

Discrete choice experiment questions

[I would now like to discuss with you the benefits and drawbacks of using disposable and reusable

Catheters]

7. What is good about your current catheter?

What do you think about single-use catheters?

What are the benefits of using a disposable or single-use catheter?

What are the drawbacks?

[in the following situations]

- At home?
- At work?
- On holiday?
- In a public place?
- Going out?
- Other?

What do you think about the idea of a re-usable catheter?

- What do you see as a benefit of re-using your catheter?
- What do you see as a drawback in re-using your catheter?

[in the following situations]

- At home?
- At work?
- On holiday?
- In a public place?
- Going out?
- Other?

- What would encourage you to use it?
- What would discourage you from using it?

Preamble: We know from previous interviews/speaking to other patients, that avoidance of infection is what matters most to everyone. We also know that other things are highly important such as:

- a comfortable insertion,
- having a catheter that is easy to use and handle,
- that comes in packaging which is easy to open,
- that your catheter is discrete,
- that you can trust your catheter/ trust in the product

As we already know all of this, we now want to explore other things around single-use catheters and re-usable catheters.

For peer review only - http://bmjopen.bmj.com/site/about/guidelines.xhtml

Together let's go through each item in this list and tell me what you think about each one? Your comments are very valuable to us.

Those relating to disposable catheters:

1. That I don't have the bother of cleaning the catheter
2. That I don't need to have special equipment for cleaning the catheter
3. That I don't have to carry used/soiled catheters around with me in my bag

Those relating to re-using catheters:

4. That I have less catheters to carry with me on holiday
5. That my catheters don't cost the NHS more than they need to/too much
6. That I don't have to store lots of catheters at home
7. That I don't have to worry that I will run out of catheters
8. That I don't have to get rid of lots of rubbish when I am out
9. That my catheters are not bad for the environment.

For peer review only

BMJ Open

Re-use of intermittent catheters: A qualitative study of IC users' perspectives

Journal:	*BMJ Open*
Manuscript ID	bmjopen-2018-021554.R1
Article Type:	Research
Date Submitted by the Author:	18-Apr-2018
Complete List of Authors:	Avery, Miriam; University of Southampton, Faculty of Health Sciences<br>Prieto, Jacqui; University of Southampton, Faculty of Health Sciences<br>Okamoto, Ikumi; University of Southampton, Faculty of Health Sciences<br>Cullen, Samantha; University of Southampton, Faculty of Health Sciences<br>Clancy, Bridget; University of Southampton, Faculty of Health Sciences<br>Moore, Katherine ; University of Alberta<br>Macaulay, Margaret; University of Southampton, Faculty of Health Sciences<br>Fader, Mandy; University of Southampton, Faculty of Health Science
**Primary Subject Heading**:	Urology
Secondary Subject Heading:	Qualitative research
Keywords:	Intermittent catheterisation, re-use, single-use, qualitative interviews

SCHOLARONE™
Manuscripts

For peer review only

TITLE PAGE

Title of the article

Re-use of intermittent catheters: A qualitative study of IC users' perspectives

Corresponding author

Miriam Avery

Address: Faculty of Health Sciences, University of Southampton, Southampton, UK

Tel.: 02381 204141

Email: M.Avery@soton.ac.uk

Co-authors

- Jacqui Prieto - Faculty of Health Sciences, University of Southampton, University Road, Southampton, SO17 1BJ, UK.
- Ikumi Okamoto – Faculty of Health Sciences, University of Southampton, University Road, Southampton, SO17 1BJ, UK.
- Samantha Cullen – Faculty of Health Sciences, University of Southampton, University Road, Southampton, SO17 1BJ, UK.
- Bridget Clancy - Faculty of Health Sciences, University of Southampton, University Road, Southampton, SO17 1BJ, UK.
- Katherine N. Moore - Faculty of Nursing, University of Alberta, Edmonton, Canada.
- Margaret Macaulay - Faculty of Health Sciences, University of Southampton, University Road, Southampton, SO17 1BJ, UK.
- Mandy Fader - Faculty of Health Sciences, University of Southampton, University Road, Southampton, SO17 1BJ, UK.

Word count (excluding title page, abstract, references, figures and tables) = 3999 (excluding box 1)

Funding

This paper refers to independent research funded by the National Institute for Health Research (NIHR) under its Programme Grants for Applied Research (PGfAR) (Grant Reference Number RP-PG-0610-10078). The views expressed in this paper are those of the authors and not necessarily those of the NHS, the NIHR or the Department of Health.

For peer review only - http://bmjopen.bmj.com/site/about/guidelines.xhtml

For peer review only

ABSTRACT

Objectives

To explore the views of intermittent catheter (IC) users regarding the advantages and disadvantages of single-use or re-use of catheters.

Design

Qualitative study with semi-structured interviews. The interviews were recorded, transcribed and analyzed thematically.

Setting

Participant's own homes in Hampshire and Dorset, UK.

Participants

A convenience sample of 39 IC users, aged 23-86 years, using IC for at least 3 months.

Results

The analysis revealed 4 main themes: concerns regarding risk of UTI; cleaning, preparation and storage; social responsibility; practicalities and location. The main concern was safety, with the fear that re-use could increase risk of urinary tract infection (UTI) compared to single-use sterile catheters. If shown to be safe then around half of participants thought they might consider reusing catheters. The practicalities of cleaning methods (extra products, time and storage) were considered potentially burdensome for re-use; but for single-use, ease of use and instant usability were advantages. Always having a catheter without fear of 'running out' was considered an advantage of re-use. Some participants were concerned about environmental impact (waste) and cost of single-use catheters. The potential for re-use was usually dependent on location. The analysis showed that often the disadvantages of single-use could be off-set by the advantages of re-use and vice versa; for example, the need to take many single-use catheters on holiday could be addressed by re-use, whilst the burden of cleaning would be obviated by single-use.

Conclusions

If shown to be safe with a practical cleaning method, some participants would find re-use an acceptable option, alongside their current single-use method. The choice to use a mixture of single-use and re-use of catheters for different activities (at home, work or holiday) could optimise the perceived advantages and disadvantages of both. The safety and acceptability of such an approach would require testing in a clinical trial.

Strengths and limitations of the study

- We believe this is the first study to explore IC users' perspectives on the advantages and disadvantages of single-use and re-use of catheters.
- Re-use is rare in the UK and the perspectives of single-use IC users about re-using catheters were therefore mostly hypothetical.
- As the primary aim of the study was to explore whether re-use of catheters would be acceptable to IC users, we may have recruited primarily individuals who were interested in issues around re-use.

For peer review only - http://bmjopen.bmj.com/site/about/guidelines.xhtml

INTRODUCTION

Intermittent catheterisation (IC) is well established management for children and adults with chronic urinary retention. The positive benefits of IC are well-known; IC users have reported relief of symptoms of frequency, urgency and incontinence, improved sleep and fewer restrictions on daily physical activities,(1, 2) and improved quality of life (QOL).(3) Although the value of IC is clear, some users describe the challenges they encounter. They report negative effects on QOL, both psychologically and socially, and urinary tract infection (UTI) remains a common problem.(1, 2) In one cross-sectional survey study, only half of the 44 participants were completely satisfied with IC.(4)

Research about IC-related issues, in particular UTI, has focussed on single-use catheters (the catheter is used once then discarded) with the development of new catheter coatings, pre-lubrication and compact designs. Re-use of uncoated catheters (the same catheter cleaned and re-used by one individual several times) has gained little attention from researchers or industry, even though single-use catheters may not be the most environmentally friendly or cost effective strategy. Catheter re-use is common in some affluent countries (e.g. Australia, Japan and Canada), but is now rare in the UK since the introduction of single-use coated catheters and subsequent changes in medical device legislation. An International guideline, prepared by the Infectious Diseases Society of America (IDSA)(5) concluded that for those living in the community and using clean self-catheterisation, the differences between sterile catheters (single-use) or reused catheters are not significant and that there is no convincing evidence that one catheter design or method is better than another in terms of UTI. This leaves clinicians with insufficient information to recommend one approach over the other.

A review of the literature was conducted and four papers were found which, to varying degrees, included user experiences of re-using IC catheters compared to single-use.(6-9) Two were qualitative studies exploring individuals' experiences of IC in general; issues raised included cost and the environmental impact of single-use compared to re-use, from a UK and US perspective.(6, 7) In two other studies,(8, 9) children with spina bifida and their families were asked about experiences of using a new hydrophilic coated single-use catheter, compared to their usual method of re-using an uncoated catheter. Some described the single-use catheter as more hygienic and felt that it was 'safer' for their child.(9) Single-use was also rated positively regarding convenience and for outings where the toilet facilities were not satisfactory.(8, 9) However, the packaging and the environmental impact of single-use catheters was raised by parents as a downside of single-use catheters.(9)

Although the potential benefits of re-use may appeal to some IC users, the potential risks and challenges are not well understood. This study is part of the National Institute for Health Research (NIHR) funded Mult*I*Cath programme (10) which aims to develop and test a catheter

For peer review only - http://bmjopen.bmj.com/site/about/guidelines.xhtml

cleaning method utilising laboratory techniques and patient panels as well as utilising interviews and surveys to determine IC user and clinician perspectives (11) regarding re-use of catheters. The purpose of this study is to explore the views of community dwelling IC users regarding the advantages and disadvantages of single-use or re-use of uncoated catheters.

METHOD

This study used a qualitative in-depth interview method. Findings from a subset of these interviews, related to the views of IC users about UTI symptoms and management, has been recently published.(12) A convenience sample of 39 IC users was recruited from 12 GP practices in Hampshire and Dorset, UK. Potential participants were identified via a computerised database search using the prescription code. GPs checked eligibility (see Box 1) and an invitation letter with a reply form, Participant Information Sheet and FREEPOST envelope were sent out by practice staff. At three weeks, reminders were sent to non-responders. Recruitment for the study continued until it was deemed data saturation had been reached. Ethics permission was granted from an NHS Research Ethics Committee (REC reference 13/LO/1511). Informed written consent was obtained from each participant and all information was kept confidential.

Box 1: Inclusion and exclusion criteria

Inclusion criteria:

- Over 18 years
- Currently using intermittent catheters for three months or more

Exclusion criteria:

- Urethral stricture or deformity
- Immune deficiency disorder (increased infection risk)
- External carer required for IC (e.g. visiting community nurse performs IC)

Data collection

All interviews were conducted face-to-face in participants' own homes by one of three trained female interviewers, including a post-doctoral qualitative researcher (SS) and two research nurses, each of whom had a least ten years' research experience (BC and MA). None of the interviewers had been in previous contact with any of the participants and they attended in a non-clinical capacity as non-judgemental listeners. A relative or carer was present for the interview if requested by the participant. A semi-structured interview schedule was used to explore experiences, values and beliefs about IC (see Appendix 1: interview schedule (Supplementary file 1)). Each interview lasted approximately 45-60 minutes. Interviews were recorded using digital audio recording equipment and then transcribed verbatim. All transcripts were anonymised with an assigned study number. The transcripts were read only by the researchers and not by participants. Field notes were made during interviews, which were used in the analysis.

For peer review only - http://bmjopen.bmj.com/site/about/guidelines.xhtml

Data analysis

Data from interviews and field notes were analysed thematically using the method described by Braun and Clarke (13) and coded using NVivo10 (QSR International) by a qualitative researcher (IO) who had not conducted the interviews. This involved moving back and forth between interview transcripts and field notes, with reference to the research literature. An initial coding framework was derived from the first 20 interview transcripts and from this the main themes emerged and sub-themes were developed. These were further refined through discussion with the wider research team and ongoing analysis of the remaining transcripts. Any newly emerged themes that did not fit into the initial themes were discussed as a team. No participants were involved in the analysis of data.

RESULTS

Participants

A total of 139 IC users were invited to take part. Seventy-four (53%) responded, of whom 42 were willing to participate. Three were ineligible, so 39 were included. The participants included 24 men and 15 women, with a mean age of 67 years (range 23-86 years) who had been using IC for a mean length of 8.5 years (range 9 months – 30 years). The frequency of IC ranged from once every second day to 10 catheterisations a day (mean 4/day). Reasons for IC included chronic urinary retention (n=20), neurological impairment (n=10) and other factors such as post-surgery or post-chemotherapy (n=9).

The majority of participants (n=34) were using a catheter once and discarding it after use. Most (n=30) were using hydrophilic coated catheters, of whom 10 used catheters with a compact design. Of the remaining five, four were currently reusing catheters designed for single-use on an on-going basis, and one had previously re-used catheters, switching to single-use 10 years ago because of convenience. The four participants who re-used catheters were all men who had been re-using for between 10 and 30 years. Their reasons for re-use were concerns about waste or cost.

Qualitative findings

The results focus on the perspectives of the participants regarding single-use and re-use of IC catheters; themes emerged about safety, burden and lifestyle. The four main themes were concerns regarding risk of UTI; cleaning, preparation and storage; social responsibility and practicalities and location. The themes and sub-themes are described below and shown in Figure 1. Those who had never re-used catheters were asked about their views but not their own experiences.

Overall, around half of the single-use participants indicated they would not want to re-use IC catheters or had major concerns about whether it would be an acceptable method. For the

For peer review only - http://bmjopen.bmj.com/site/about/guidelines.xhtml

remaining participants, the potential acceptability of re-use was very much dependent on the safety and efficacy of the cleaning regimen and whether there was an increased risk of infection, as well as factors such as convenience and practicalities (Figure 1).

1. Concerns regarding risk of UTI

1.1 Safety: Fears about increasing risk of UTI

The question of UTI risk was the main concern raised by many single-users when asked about re-using catheters. This was the case for those who had suffered UTIs and others who had not and for men and women participants. They described their concerns about infection as their biggest worry and for some it was the only issue they had when considering re-use.

> *Infection [concerns me about re-using catheters] definitely. Especially as I've not had any infection, I think I'm doing something right, if you get me. (Participant 22, female, single-user)*

> *My honest opinion [about reusing] is I can understand why they're trying to do it, it's they're trying to save money. But in my opinion there's always a chance that that's going to go wrong and I'm the one who's gonna end up having a water infection or something like that. (Participant 13, male, single-user)*

Many perceived that using single-use catheters meant they had less chance of getting an infection and they felt that the single-use method was more 'clean' and 'sterile'.

> *[Single-use catheters have] less chance on infection, you can dispose of it, get rid of it. (Participant 40, male, single-user)*

However, the four participants who were re-using were not deterred by fears about infection.

1.2 Necessity of safe cleaning method

Related to the fears described above, some single-users described the importance of an effective catheter cleaning method as a pre-requisite for re-use.

> *[It is important] that you follow all the steps that you are supposed to. Because if you don't then it might not be sterile. Because I don't really know what it, what those effective ways would be. (Participant 16, female, single-user)*

For peer review only - http://bmjopen.bmj.com/site/about/guidelines.xhtml

2 Cleaning, preparation, and storage

2.1 Concerns about a complicated/time-consuming cleaning procedure

Many single-users thought that the cleaning procedure would be complicated and were concerned about the amount of work and time involved. Some did not want to be bothered with it and thought it could be inconvenient and potentially messy.

> *….but I mean if you had to go to all the bother of washing a catheter, I don't know how you'd do it to be quite honest, to get it really sterile. (Participant 29, female, single-user)*

Some single-users mentioned that they needed to know more about the cleaning method before they could comment on re-use. It appeared, however, that all four of the catheter re-users were conducting a very simple catheter cleaning procedure, typically using only tap water to rinse out used catheters.

> *Holding it under the tap [running it under tap water], and then I put it right back in the pouch, and then it's as clean as a whistle. […] (Participant 19, male, re-user)*

2.2 Concerns about storage and supply of catheters

The participants who would consider re-use of catheters thought it could save space in the home. However, some suggested that finding space to store extra equipment for cleaning catheters would be a problem. One participant, with a complex long-term health condition that required other equipment, was concerned about this due to the amount of equipment she already had.

> *My bathroom is already clogged full of all my bulky stuff, I already have my sort of like bag that hangs up for flushing my bowels out.[…] I think that I'd want it to not be too bulky. (Participant 11, female, single-user)*

In terms of catheter supply, although some participants were very happy with their current supply arrangements, for others the prospect of a supply shortage was a drawback of single-use catheters. Some participants thought they would not need to worry about running out if they could re-use them.

For peer review only - http://bmjopen.bmj.com/site/about/guidelines.xhtml

Wouldn't have to keep running up the chemist [if I used reusable catheters]. You'd know you've always got one. You would know there's always one there. (Participant 7, female, single-user)

3 Social responsibility

3.1 Reducing costs to NHS

Many participants expressed their concerns or could see the issues around the financial cost of their catheters for the National Health Service (NHS) and some suggested that they would consider re-use to contribute to reducing such cost.

I think the GP's surgery pays for [my catheter] but I found out how much it was last week and I was shocked, really shocked actually. I feel guilty about that. (Participant 15, female, single-user)

Some described the potential cost-saving as the only benefit of re-use and noted that the benefit was not to them directly. Two participants questioned whether there would be any saving, with one considering that it may cost more due to the potential for increased infections.

3.2 Reducing waste and the impact on the environment

Several participants felt that re-using catheters would reduce waste and, for that reason, might be more environmentally friendly.

The good thing about re-using would be that it would cut down the amount of waste. If I go through [single-use catheters] every 6 weeks that is a mountain of catheters every year I get though. So I think that is the main benefit really. (Participant 38, female, single-user)

A participant who was re-using indicated that not disposing of the catheter after each use alleviated the worry about contributing to landfill waste. Another re-user described how reducing waste was the main motive to start reusing their catheters.

I hate wasting anything, and so that's all built into why I use the catheter more than once. (Participant 25, male, re-user)

For peer review only - http://bmjopen.bmj.com/site/about/guidelines.xhtml

4 Practicalities and Location

4.1 Freedom and a normal life

Most single-users appreciated their current catheters as they were convenient, comfortable and easy to use, with some describing them as giving freedom and a normal life; IC had become part of life, a natural thing like cleaning teeth.

> *I love that [my catheter] is compact. They are just so convenient to use. No fuss. It doesn't impact on your normal daily lifestyle. They are great for travelling. That's what I love about my catheters. And it gives me a normal life; otherwise I would probably have to be at home more really. (Participant 38, female, 48)*

The participants were often familiar with the design of their current single-use catheter and described how it suited them personally, although this may not directly relate to single-use or reusability.

When considering re-use, the participants discussed the locations where they would be more likely to re-use their catheters; they often compared the practicalities of re-use inside and outside the home as well as issues around travel.

4.2 Travel

The compact size of single-use catheters was appreciated by participants because they were easy to carry individually. However, for holidays, the need to take a large supply was a hindrance. In this situation, participants noted that re-use of catheters could be a more practical option.

One participant valued this aspect of reusing from his experience.

> *If I take [my catheters], I'll take 10 for the month or something. If I take the other one I've got to take 120. […] I did a trip around Australia once with my family and we were away 30 days or a month and the difference would have been huge. I pretty much would have needed a suitcase just for that. So that's always been a major issue. (Participant 37, male, re-user)*

However, several single-users were concerned about carrying the cleaning equipment on holiday.

For peer review only - http://bmjopen.bmj.com/site/about/guidelines.xhtml
For peer review only

4.3 Carrying used catheters

Many participants appreciated the portability and ease of disposal of the single-use catheters. Some single-users, therefore, wondered what they would have to do with the used catheters if they catheterised when they were out.

> *If you are going out for the day. You'd use your catheter in the morning; I don't want to take that one around with me. I don't know how I could. (Participant 41, male, single-user)*

However, one of the re-users emphasised the possible flexibility of reusing their catheter, as they could also be thrown away after a single use if needed.

> *You can always throw it away and you've only done the same as you've done with a non-re-usable one. So if you find yourself out and about and it's not as hygienic as you'd have liked; […] I will tend to use it once and throw it away under those circumstances. (Participant 37, male, 50, single-user)*

4.4 Discretion

The majority of single-users appreciated the compact packaging and discreetness of their current catheters. Many were concerned they would not have these advantages if they re-used catheters and the lack of discreetness would restrict them.

> *[Single-use catheters are] just quick really, it doesn't take me any longer than somebody else going into the toilet, not much longer at all so it's that easy. If you have to start cleaning it after using it….while you're out, it then gets a bit [much]. And it's obviously difficult to do it in a public toilet as well. (Participant 3, female, single-user)*

While some said that they would be happy to re-use at home, others expressed their concern, as they did not want visitors to see used catheters or the cleaning equipment.

4.5 Choice of catheters – mixed use

Among those who viewed re-use as potentially acceptable, most said they would value having both types of catheters to choose from depending on the situation.

> *Important stuff would be that when I'm doing it at home it's not generally a problem. If I know what I've got to do I will do it. It's*

For peer review only - http://bmjopen.bmj.com/site/about/guidelines.xhtml

> *knowing that when you are going away, you don't know where you are going to, […} is that equipment there? For me to be able to do it? (Participant 1, male, single-user)*

> *…..I wouldn't want to be forced to have just re-used catheters. But in most circumstances I would choose to use it accept for when I'm travelling in which case I would definitely want to be using the disposable ones. And I think generally speaking if you are away you don't want all the faff. (Participant 38, female, single-user)*

DISCUSSION

We believe this is the first study to explore IC users' perspectives on the advantages and disadvantages of single-use and re-use of catheters. The findings highlight the complexity of factors involved and show the variation of views amongst IC users; for some, re-use is not an acceptable option and for others it could be acceptable, but robust evidence is needed regarding safety, and the cleaning method must be shown to be effective and user-friendly.

Concerns regarding risk of UTI

For IC users to consider re-use they must first be satisfied that it is safe to do so; this was the crucial factor for many. In fact, around half of participants in this study said they would not consider re-use or had major concerns about it, mainly due to fears about infection. A guideline for the diagnosis, treatment and prevention of catheter associated UTI (IDSA) concluded that there have not been enough robust studies to provide evidence regarding risk of UTI when comparing re-use of catheters to single-use catheters.(5) This emphasises the need for a randomised controlled trial to properly examine this.

Cleaning, preparation and storage

The development of a cleaning method that is practical for IC users was an issue raised by participants. Concerns were voiced about the number of cleaning items requiring storage and the potential burden of doing the cleaning as well as the effectiveness of the cleaning method. Others have suggested that the cleaning method could be a burden when re-using catheters (14) which could in turn impair both independence and adherence;(15) Chick et al (2015) found that some families in their study preferred the convenience of the one-step process for single-use catheters.(8) It is therefore important that the cleaning method is developed with IC users alongside rigorous laboratory testing.(16)

Social responsibility

Despite participants' concerns about re-use, they recognised some advantages. Many thought there could be cost savings for the NHS and a positive environmental impact and some were currently re-using, or would consider re-using, for these reasons. Similar findings were reported by Kelly in which 16 individuals were interviewed about their experiences and concerns about IC

For peer review only - http://bmjopen.bmj.com/site/about/guidelines.xhtml

use. Financial burden to the health service and environmental impact were raised by 63% (10/16) and 38% (6/16) of the participants respectively; the authors suggested this reflects the greater awareness of environmental issues in society.(6) Although several of the participants in this study felt that re-use of catheters would be more environmentally friendly, this is a complex issue dependent on the method used for cleaning; there is a need for knowledge to be gained on environmental issues.

Four distinct modelling studies on cost effectiveness using different IC catheters and methods have been published. Both Neovius(17) and Bermingham(18) compared re-use with single-use catheters. In a conference abstract, Neovius concluded that re-use of PVC catheters was not cost effective compared to single-use coated catheters,(17) whereas Bermingham indicated that re-use could be more cost effective than single-use catheters but that additional evidence was required before any practice changes could be recommended.(18) Clark and colleagues(19) concluded that single-use coated rather than single-use uncoated catheters were more cost effective when outcomes were calculated over a patient's lifetime. Neovius and Lundquist(20) indicated that patients would be willing to pay out of pocket for a hypothetical catheter that would reduce/prevent UTIs. The probabilistic decision model of these studies regarding risk of UTI limits the interpretation of the results and indicates, as Bermingham stated, that further robust evidence is required.

Practicalities and location

Although many of the participants would consider catheter re-use, willingness to do so varied between individuals based on lifestyle and circumstances. The need for discretion was of great concern and was closely linked to location; some participants would only consider re-using catheters when they were at home. The literature also confirms that location, and specifically whether at home or away from home, has a bearing on how people feel about re-use.(8, 9) This highlights the need for a flexible approach to IC methods, which may be achieved by giving IC users the option to use both catheter types to suit their lifestyle and preferences.

Many of the perceived disadvantages of re-use are off-set by advantages of single-use and vice versa (see Figure 1). For example, some IC users were concerned about running out of their single-use catheters, and the ability to re-use their catheter would mean they always have a usable catheter. Others emphasised the benefit of using single-use catheters when in public places and the difficulties of re-use when away from home reinforcing the potential benefit of mixed use. Furthermore, if re-use of catheters was more commonplace, efforts could be made to design and manufacture discreet, re-usable versions in the same way as for single-use.

Limitations

As the primary aim of the study was to explore whether reusing catheters would be acceptable to IC users in preparation for a randomised controlled trial, and this was stated in the participant information sheet, we may have recruited primarily individuals who were interested in issues around re-use. Therefore, findings may not be representative of all UK IC users. Furthermore,

For peer review only - http://bmjopen.bmj.com/site/about/guidelines.xhtml

the perspectives of single-use IC users about re-using catheters were hypothetical and not based on their actual experiences. The inclusion of questions about advantages and disadvantages of both methods may have influenced participants responses and the researcher's presence during the data collection might have elicited socially desirable responses; however, the researcher presented as an impartial observer during the data collection and every effort was made to keep the interviews balanced.

Implications for future research/clinicians/policy makers

This study has shown that some IC users would find re-use of catheters acceptable, but there is a need to develop a rigorous cleaning method for IC catheters which is effective and user-friendly. If shown to be safe, then efforts could be made to develop user-friendly catheter designs, e.g. compact designs, with an efficient cleaning method.

CONCLUSION

This study revealed that if shown to be safe with a practical cleaning method, some participants would find re-use of catheters for some of the time an acceptable option. The choice to use a mixture of single-use and re-use of catheters for different activities (at home, work or holiday) could optimise the advantages and disadvantages of both methods. With environmental issues and cost high on the public agenda, some IC users would advocate that if re-use is shown to be safe and acceptable then it could be made available as an option. The safety and acceptability of such an approach would require testing in a clinical trial.

Acknowledgements

We would like to thank all the interviewees who agreed to take part and share their experiences.

Contributors

MF was the chief investigator and was primarily responsible for the original grant application. MF, JP, MM, SC and MA were involved in designing the study and methods. JP led the overall study team. SC collected the majority of the data. BC and MA contributed to the data collection. IO led the analysis and IO and MA were involved in interpretation of the qualitative data and wrote draft manuscripts. IO, JP, MA, KNM, MM and MF wrote drafts and/or critically revised the manuscripts.

Patient consent

Detail has been removed from this case description/these case descriptions to ensure anonymity.

Competing interests

None declared

For peer review only - http://bmjopen.bmj.com/site/about/guidelines.xhtml

Data sharing statement

No additional data is available.

REFERENCE LIST

1. McConville A. Patients' experiences of clean intermittent catheterisation. *NTplus* 2002;98(4):55-56.
2. Shaw C, Logan K, Webber I, et al. Effect of clean intermittent self-catheterization on quality of life: a qualitative study. *J Adv Nurs* 2008;61(6):641-50.
3. Kessler TM, Ryu G, Burkhard FC. Clean intermittent self-catheterization: a burden for the patient? *Neurourol Urodyn* 2009;28:18-21.
4. Bolinger R, Engberg S. Barriers, complications, adherence, and self-reported quality of life for people using clean intermittent catheterization. *J Wound Ostomy Continence Nurs* 2013;40(1):83-89.
5. Hooton TM, Bradley SF, Cardenas DD, et al. Diagnosis, prevention, and treatment of catheter-associated urinary tract Infection in adults: 2009 international clinical practice guidelines from the Infectious Diseases Society of America. *Clin Infect Dis* 2010;50:625-63.
6. Kelly L, Spencer S, Barrett G. Using intermittent self-catheters: experiences of people with neurological damage to their spinal cord. *Disabil Rehabil* 2014;36(3):220-226.
7. Wilde MH, Brasch J, Zhang Y. A qualitative descriptive study of self-management issues in people with long-term intermittent urinary catheters. *J Adv Nurs* 2011;67(6):1254-63.
8. Chick HE, Hunter KF, Moore KN. Parent and child experiences using a hydrophilic or reused PVC catheter for intermittent catheterisation. *J Clin Nurs* 2012;22:513-520.
9. Kiddoo D, Sawatzky B, Bascu C-D, et al. Randomized crossover trial of single use hydrophilic coated versus multiple use polyvinylchloride catheters for intermittent catheterization to determine incidence of urinary infection. *J Urol* 2015;194:174-179.
10. Fader M. Development and clinical trial of a mixed (multi/single-use) catheter management package for users of intermittent catheterisation. *National Institute for Health Research (NIHR) under its Programme Grants for Applied Research (PGfAR)*. Programme (Grant Reference Number RP-PG-0610-10078), 2013
11. McClurg D, Coyle J, Long A, et al. A two phased study on health care professionals' perceptions of single or multi-use of intermittent catheters. *Int J Nurs Stud* 2017;72:83-90.
12. Okamoto I, Prieto J, Avery M, et al. Intermittent catheter users' symptom identification, description and management of urinary tract infection: a qualitative study. *BMJ Open* 2017;7: e016453. doi:10.1136/bmjopen-2017-016453.
13. Braun V, Clarke V. Using thematic analysis in psychology. *Qual research psychol* 2006;3(2):77-101.
14. Woodward S. Improving quality of life for men using intermittent self-catheterisation. *British Journal of Neuroscience Nursing.* 2013;9(3):114-119.
15. Dean G. Are Single Use Catheters Worth the Expense? *J Urol* 2015;194(1):12-3.
16. Wilks S, Morris N, Delgado D, et al. Development of an effective and acceptable cleaning method to allow safe re-use of plain, uncoated catheters for intermittent catheterization. *Neurourol Urodyn* (Abstract 566) 2016;4:449-450.
17. Neovius K, Hakansson M, Lundqvist T. Cost consequences of single-use and re-use of urinary catheters among patients performing daily intermittent catheterisation (Abstract PMD43). *Value in Health* 2014;18:A351.

For peer review only

For peer review only - http://bmjopen.bmj.com/site/about/guidelines.xhtml

18. Bermingham SL, Hodgkinson S, Wright S, et al. Intermittent self catheterisation with hydrophilic, gel reservoir, and non-coated catheters: a systematic review and cost effectiveness analysis. *BMJ* 2013; 346:e8639
19. Clark JF, Mealing SJ, Scott DA, et al. A cost-effectiveness analysis of long-term intermittent catheterisation with hydrophilic and uncoated catheters. *Spinal Cord* 2016;54(1):73-77.
20. Neovius KE, Lundqvist T. CIC users' preference for catheters reducing the UTI frequency (Abstract PUK14). *Value in Health* 2014;17:A468.

Figure Legend

Figure 1: Themes, sub-themes and coding from analysis of qualitative interviews

For peer review only

For peer review only - http://bmjopen.bmj.com/site/about/guidelines.xhtml

Main theme	Sub-theme	Codes			
		Re-use		Single-use	
		Advantages	Disadvantages	Advantages	Disadvantages
**1. Concerns regarding risk of UTI**	1.1 Safety: fears about increasing risk of UTI		Concern about hygiene and perceived link to UTI	Considered less likely to cause infection<br>Sterile	
	1.2 Necessity of safe cleaning		Cleaning and preparation		
**2. Cleaning, preparation and storage**	2.1 Concerns about a complicated, time-consuming cleaning procedure		Cleaning and preparation		
	2.2 Concerns about storage/supply of catheters	Always having one<br>No storage problem	Storing equipment<br>Concern use-by-date could expire	Good delivery system/good relationship with supplier	Shortage of supply/not good delivery service
**3. Social responsibility**	3.1 Reducing costs to NHS	Cost			Cost
	3.2 Reducing waste/impact on environment	Environment			Environment and waste
**4. Practicalities and location**	4.1 Freedom and a normal life		Limiting lifestyle	Instantly usable or easy to use and to dispose<br>No pain and easy to use<br>Gives freedom/normal life<br>More independence	
	4.2 Travel	Less to carry when travelling	Taking cleaning equipment on holiday		Not easy to carry on holiday
	4.3 Carrying used catheters		Carrying used catheters		
	4.4 Discretion		Not discreet	Easy to carry and discreteness<br>Ready to use<br>Easy to use	Don't like to dispose used catheters in public toilets<br>Not easy to carry around
	4.5 Choice of catheters – mixed use	Emerged from interviews			

Figure 1: Themes, sub-themes and coding from analysis of qualitative interviews

108x60mm (300 x 300 DPI)

review only

MultICath Module 2 Schedule for iteration 3

Background questions

1. Experience of intermittent catheterisation:

How long have you been using intermittent catheterisation?

Do you self-catheterise? Or does somebody help you?

How many times per day do you catheterise?

What catheter do you use?

Do you catheterise:

At home only?

When you are out?

If yes, when you are out what kind of catheter do you use, is it the same one as for home use?

Beliefs and practices re IC routine

2. Beliefs and concerns about IC rituals, catheters, infection and sterility

What steps do you take before you do IC?

What is the reason you take these steps? (Probe without leading for whether or not they value sterility)

Is your IC routine clean or sterile?

What is sterile to you? (prompt – how is it different to clean?) What do you mean by clean?

Experience of urine infection

3. During your time using intermittent catheterisation have you ever experienced a urine infection?

If yes, how often would you say you have a urine infection?

Can you recall the last time you had a UTI? What happened?

4. What it feels like to have a urine infection:

Can you describe how you feel when you think you may be developing a urine infection?

Does anything happen beforehand when you think one is coming on?

For peer review only - http://bmjopen.bmj.com/site/about/guidelines.xhtml

How do you think your infection came about? Prompt (Where do you think the germs come from when you get a UTI?)

What do you do to prevent an infection?

What advice do you get/have you had from your health care professional to prevent infections?

Complete the table – UTI signs and symptoms: Where patients name a symptom or sign check with them the terminology and that the patient is happy that it sits within a category on the table. We want to understand the terminology that patients use to describe UTI symptoms.

Looking at the symptoms listed here, are these the right things that describe a urine infection from your experience? Is anything missing?

5. Seeking help:

When you feel you may have a urine infection, what do you do about it?

Prompt: antibiotics

At what point do you seek help from others?

Who do you seek help from? And what do you do?

Cleaning questions

6. Methods of cleaning:

Have you ever re-used your catheter?

If Yes, under what circumstances? Did you clean it after its first use? How did you clean it? How did you store it between uses?

What is/would be acceptable to you?

If No, Have you ever thought about re-using your catheters?

If you were offered a method of cleaning and re-using your catheters instead of using a new one each time, what kinds of things would be important to you? [Prompts: How to clean; Where to clean; Where to store between uses]

What kind of cleaning regime is acceptable to you? [Prompts: what cleaning methods and products, what about storage storage/travelling, and lubrication?]

What would worry you about re-using your catheter? [Prompts: cleaning in a public loo, being at work, storing and transporting]

What would be good about re-using?

For peer review only - http://bmjopen.bmj.com/site/about/guidelines.xhtml

Discrete choice experiment questions

[I would now like to discuss with you the benefits and drawbacks of using disposable and reusable Catheters]

7. What is good about your current catheter?

What do you think about single-use catheters?

What are the benefits of using a disposable or single-use catheter?

What are the drawbacks?

[in the following situations]

- At home?
- At work?
- On holiday?
- In a public place?
- Going out?
- Other?

What do you think about the idea of a re-usable catheter?

- What do you see as a benefit of re-using your catheter?
- What do you see as a drawback in re-using your catheter?

[in the following situations]

- At home?
- At work?
- On holiday?
- In a public place?
- Going out?
- Other?

- What would encourage you to use it?
- What would discourage you from using it?

Preamble: We know from previous interviews/speaking to other patients, that avoidance of infection is what matters most to everyone. We also know that other things are highly important such as:

- a comfortable insertion,
- having a catheter that is easy to use and handle,
- that comes in packaging which is easy to open,
- that your catheter is discrete,
- that you can trust your catheter/ trust in the product

As we already know all of this, we now want to explore other things around single-use catheters and re-usable catheters.

For peer review only - http://bmjopen.bmj.com/site/about/guidelines.xhtml

Together let's go through each item in this list and tell me what you think about each one? Your comments are very valuable to us.

Those relating to disposable catheters:

1. That I don't have the bother of cleaning the catheter
2. That I don't need to have special equipment for cleaning the catheter
3. That I don't have to carry used/soiled catheters around with me in my bag

Those relating to re-using catheters:

4. That I have less catheters to carry with me on holiday
5. That my catheters don't cost the NHS more than they need to/too much
6. That I don't have to store lots of catheters at home
7. That I don't have to worry that I will run out of catheters
8. That I don't have to get rid of lots of rubbish when I am out
9. That my catheters are not bad for the environment.

For peer review only

For peer review only - http://bmjopen.bmj.com/site/about/guidelines.xhtml

COREQ (COnsolidated criteria for REporting Qualitative research) Checklist

A checklist of items that should be included in reports of qualitative research. You must report the page number in your manuscript where you consider each of the items listed in this checklist. If you have not included this information, either revise your manuscript accordingly before submitting or note N/A.

Topic	Item No.	Guide Questions/Description	Reported on Page No.
**Domain 1: Research team and reflexivity**			
Personal characteristics			
Interviewer/facilitator	1	Which author/s conducted the interview or focus group?	
Credentials	2	What were the researcher's credentials? E.g. PhD, MD	
Occupation	3	What was their occupation at the time of the study?	
Gender	4	Was the researcher male or female?	
Experience and training	5	What experience or training did the researcher have?	
Relationship with participants			
Relationship established	6	Was a relationship established prior to study commencement?	
Participant knowledge of the interviewer	7	What did the participants know about the researcher? e.g. personal goals, reasons for doing the research	
Interviewer characteristics	8	What characteristics were reported about the inter viewer/facilitator? e.g. Bias, assumptions, reasons and interests in the research topic	
**Domain 2: Study design**			
Theoretical framework			
Methodological orientation and Theory	9	What methodological orientation was stated to underpin the study? e.g. grounded theory, discourse analysis, ethnography, phenomenology, content analysis	
Participant selection			
Sampling	10	How were participants selected? e.g. purposive, convenience, consecutive, snowball	
Method of approach	11	How were participants approached? e.g. face-to-face, telephone, mail, email	
Sample size	12	How many participants were in the study?	
Non-participation	13	How many people refused to participate or dropped out? Reasons?	
Setting			
Setting of data collection	14	Where was the data collected? e.g. home, clinic, workplace	
Presence of non-participants	15	Was anyone else present besides the participants and researchers?	
Description of sample	16	What are the important characteristics of the sample? e.g. demographic data, date	
Data collection			
Interview guide	17	Were questions, prompts, guides provided by the authors? Was it pilot tested?	
Repeat interviews	18	Were repeat inter views carried out? If yes, how many?	
Audio/visual recording	19	Did the research use audio or visual recording to collect the data?	
Field notes	20	Were field notes made during and/or after the inter view or focus group?	
Duration	21	What was the duration of the inter views or focus group?	
Data saturation	22	Was data saturation discussed?	
Transcripts returned	23	Were transcripts returned to participants for comment and/or	

For peer review only

Topic	Item No.	Guide Questions/Description	Reported on Page No.
		correction?	
**Domain 3: analysis and findings**			
Data analysis			
Number of data coders	24	How many data coders coded the data?	
Description of the coding tree	25	Did authors provide a description of the coding tree?	
Derivation of themes	26	Were themes identified in advance or derived from the data?	
Software	27	What software, if applicable, was used to manage the data?	
Participant checking	28	Did participants provide feedback on the findings?	
Reporting			
Quotations presented	29	Were participant quotations presented to illustrate the themes/findings? Was each quotation identified? e.g. participant number	
Data and findings consistent	30	Was there consistency between the data presented and the findings?	
Clarity of major themes	31	Were major themes clearly presented in the findings?	
Clarity of minor themes	32	Is there a description of diverse cases or discussion of minor themes?	

Developed from: Tong A, Sainsbury P, Craig J. Consolidated criteria for reporting qualitative research (COREQ): a 32-item checklist for interviews and focus groups. *International Journal for Quality in Health Care*. 2007. Volume 19, Number 6: pp. 349 – 357

Once you have completed this checklist, please save a copy and upload it as part of your submission. DO NOT include this checklist as part of the main manuscript document. It must be uploaded as a separate file.

For peer review only

BMJ Open

Re-use of intermittent catheters: A qualitative study of IC users' perspectives

Journal:	*BMJ Open*
Manuscript ID	bmjopen-2018-021554.R2
Article Type:	Research
Date Submitted by the Author:	18-Jun-2018
Complete List of Authors:	Avery, Miriam; University of Southampton, Faculty of Health Sciences<br>Prieto, Jacqui; University of Southampton, Faculty of Health Sciences<br>Okamoto, Ikumi; University of Southampton, Faculty of Health Sciences<br>Cullen, Samantha; University of Southampton, Faculty of Health Sciences<br>Clancy, Bridget; University of Southampton, Faculty of Health Sciences<br>Moore, Katherine ; University of Alberta<br>Macaulay, Margaret; University of Southampton, Faculty of Health Sciences<br>Fader, Mandy; University of Southampton, Faculty of Health Science
**Primary Subject Heading**:	Urology
Secondary Subject Heading:	Qualitative research
Keywords:	Intermittent catheterisation, re-use, single-use, qualitative interviews

SCHOLARONE™ Manuscripts

For peer review only

TITLE PAGE

Title of the article

Re-use of intermittent catheters: A qualitative study of IC users' perspectives

Corresponding author

Miriam Avery

Address: Faculty of Health Sciences, University of Southampton, Southampton, UK

Tel.: 02381 204141

Email: M.Avery@soton.ac.uk

Co-authors

- Jacqui Prieto - Faculty of Health Sciences, University of Southampton, University Road, Southampton, SO17 1BJ, UK.
- Ikumi Okamoto – Faculty of Health Sciences, University of Southampton, University Road, Southampton, SO17 1BJ, UK.
- Samantha Cullen – Faculty of Health Sciences, University of Southampton, University Road, Southampton, SO17 1BJ, UK.
- Bridget Clancy - Faculty of Health Sciences, University of Southampton, University Road, Southampton, SO17 1BJ, UK.
- Katherine N. Moore - Faculty of Nursing, University of Alberta, Edmonton, Canada.
- Margaret Macaulay - Faculty of Health Sciences, University of Southampton, University Road, Southampton, SO17 1BJ, UK.
- Mandy Fader - Faculty of Health Sciences, University of Southampton, University Road, Southampton, SO17 1BJ, UK.

Word count (excluding title page, abstract, references, figures and tables) = 4093 (excluding box 1)

Funding

This paper refers to independent research funded by the National Institute for Health Research (NIHR) under its Programme Grants for Applied Research (PGfAR) (Grant Reference Number RP-PG-0610-10078). The views expressed in this paper are those of the authors and not necessarily those of the NHS, the NIHR or the Department of Health.

For peer review only - http://bmjopen.bmj.com/site/about/guidelines.xhtml

ABSTRACT

Objectives

To explore the views of intermittent catheter (IC) users regarding the advantages and disadvantages of single-use or re-use of catheters.

Design

Qualitative study with semi-structured interviews. The interviews were recorded, transcribed and analyzed thematically.

Setting

Participant's own homes in Hampshire and Dorset, UK.

Participants

A convenience sample of 39 IC users, aged 23-86 years, using IC for at least 3 months.

Results

The analysis revealed 4 main themes: concerns regarding risk of UTI; cleaning, preparation and storage; social responsibility; practicalities and location. The main concern was safety, with the fear that re-use could increase risk of urinary tract infection (UTI) compared to single-use sterile catheters. If shown to be safe then around half of participants thought they might consider re-using catheters. The practicalities of cleaning methods (extra products, time and storage) were considered potentially burdensome for re-use; but for single-use, ease of use and instant usability were advantages. Always having a catheter without fear of 'running out' was considered an advantage of re-use. Some participants were concerned about environmental impact (waste) and cost of single-use catheters. The potential for re-use was usually dependent on location. The analysis showed that often the disadvantages of single-use could be off-set by the advantages of re-use and vice versa; for example, the need to take many single-use catheters on holiday could be addressed by re-use, whilst the burden of cleaning would be obviated by single-use.

Conclusions

If shown to be safe with a practical cleaning method, some participants would find re-use an acceptable option, alongside their current single-use method. The choice to use a mixture of single-use and re-use of catheters for different activities (at home, work or holiday) could optimise the perceived advantages and disadvantages of both. The safety and acceptability of such an approach would require testing in a clinical trial.

Strengths and limitations of the study

- We believe this is the first study to explore IC users' perspectives on the advantages and disadvantages of single-use and re-use of catheters.
- Re-use is rare in the UK and the perspectives of single-use IC users about re-using catheters were therefore mostly hypothetical.
- As the primary aim of the study was to explore whether re-use of catheters would be acceptable to IC users, we may have recruited primarily individuals who were interested in issues around re-use.

For peer review only - http://bmjopen.bmj.com/site/about/guidelines.xhtml

INTRODUCTION

Intermittent catheterisation (IC) is well established management for children and adults with chronic urinary retention. The positive benefits of IC are well-known; IC users have reported relief of symptoms of frequency, urgency and incontinence, improved sleep and fewer restrictions on daily physical activities,(1, 2) and improved quality of life (QOL).(3) Although the value of IC is clear, some users describe the challenges they encounter. They report negative effects on QOL, both psychologically and socially, and urinary tract infection (UTI) remains a common problem.(1, 2) In one cross-sectional survey study, only half of the 44 participants were completely satisfied with IC.(4)

Research about IC-related issues, in particular UTI, has focussed on single-use catheters (the catheter is used once then discarded) with the development of new catheter coatings, pre-lubrication and compact designs. Re-use of uncoated catheters (the same catheter cleaned and re-used by one individual several times) has gained little attention from researchers or industry, even though single-use catheters may not be the most environmentally friendly or cost effective strategy. Catheter re-use is common in some affluent countries (e.g. Australia, Japan and Canada), but is now rare in the UK since the introduction of single-use coated catheters and subsequent changes in medical device legislation. An International guideline, prepared by the Infectious Diseases Society of America (IDSA)(5) concluded that for those living in the community and using clean self-catheterisation, the differences between sterile catheters (single-use) or reused catheters are not significant and that there is no convincing evidence that one catheter design or method is better than another in terms of UTI. This leaves clinicians with insufficient information to recommend one approach over the other.

A review of the literature was conducted and four papers were found which, to varying degrees, included user experiences of re-using IC catheters compared to single-use.(6-9) Two were qualitative studies exploring individuals' experiences of IC in general; issues raised included cost and the environmental impact of single-use compared to re-use, from a UK and US perspective.(6, 7) In two other studies,(8, 9) children with spina bifida and their families were asked about experiences of using a new hydrophilic coated single-use catheter, compared to their usual method of re-using an uncoated catheter. Some described the single-use catheter as more hygienic and felt that it was 'safer' for their child.(9) Single-use was also rated positively regarding convenience and for outings where the toilet facilities were not satisfactory.(8, 9) However, the packaging and the environmental impact of single-use catheters was raised by parents as a downside of single-use catheters.(9)

Although the potential benefits of re-use may appeal to some IC users, the potential risks and challenges are not well understood. This study is part of the National Institute for Health Research (NIHR) funded Mult*I*Cath programme (10) which aims to develop and test a catheter

For peer review only - http://bmjopen.bmj.com/site/about/guidelines.xhtml

cleaning method utilising laboratory techniques and patient panels as well as utilising interviews and surveys to determine IC user and clinician perspectives (11) regarding re-use of catheters. The purpose of this study is to explore the views of community dwelling IC users regarding the advantages and disadvantages of single-use or re-use of uncoated catheters.

METHOD

This study used a qualitative in-depth interview method. Findings from a subset of these interviews, related to the views of IC users about UTI symptoms and management, has been recently published.(12) A convenience sample of 39 IC users was recruited from 12 GP practices in Hampshire and Dorset, UK. Potential participants were identified via a computerised database search using the prescription code. GPs checked eligibility (see Box 1) and an invitation letter with a reply form, Participant Information Sheet and FREEPOST envelope were sent out by practice staff. At three weeks, reminders were sent to non-responders. Recruitment for the study continued until it was deemed data saturation had been reached. Ethics permission was granted from an NHS Research Ethics Committee (REC reference 13/LO/1511). Informed written consent was obtained from each participant and all information was kept confidential.

Box 1: Inclusion and exclusion criteria

Inclusion criteria:

- Over 18 years
- Currently using intermittent catheters for three months or more

Exclusion criteria:

- Urethral stricture or deformity
- Immune deficiency disorder (increased infection risk)
- External carer required for IC (e.g. visiting community nurse performs IC)

Data collection

All interviews were conducted face-to-face in participants' own homes by one of three trained female interviewers, including a post-doctoral qualitative researcher (SS) and two research nurses, each of whom had a least ten years' research experience (BC and MA). None of the interviewers had been in previous contact with any of the participants and they attended in a non-clinical capacity as non-judgemental listeners. A relative or carer was present for the interview if requested by the participant. A semi-structured interview schedule was used to explore experiences, values and beliefs about IC (see Appendix 1: interview schedule (Supplementary file 1)). Each interview lasted approximately 45-60 minutes. Interviews were recorded using digital audio recording equipment and then transcribed verbatim. All transcripts were anonymised with an assigned study number. The transcripts were read only by the researchers and not by participants. Field notes were made during interviews, which were used in the analysis.

For peer review only - http://bmjopen.bmj.com/site/about/guidelines.xhtml

Data analysis

Data from interviews and field notes were analysed thematically using the method described by Braun and Clarke (13) and coded using NVivo10 (QSR International) by a qualitative researcher (IO) who had not conducted the interviews. This involved moving back and forth between interview transcripts and field notes, with reference to the research literature. An initial coding framework was derived from the first 20 interview transcripts and from this the main themes emerged and sub-themes were developed. These were further refined through discussion with the wider research team and ongoing analysis of the remaining transcripts. Any newly emerged themes that did not fit into the initial themes were discussed as a team. No participants were involved in the analysis of data.

Patient and public involvement

The research question was derived from a consumer-clinician priority setting exercise by the James Lind Alliance in 2008. Patient and Public Involvement (PPI) representatives (two women and one man) were members of the Mult*I*Cath Project Management Group and supported the Programme to ensure that participant experience was considered at each stage of the research. They contributed to the development of the study design, processes and materials. Bladder & Bowel UK, a key incontinence consumer organisation, is a collaborator on the Mult*I*Cath programme.

RESULTS

Participants

A total of 139 IC users were invited to take part. Seventy-four (53%) responded, of whom 42 were willing to participate. Three were ineligible, so 39 were included. The participants included 24 men and 15 women, with a mean age of 67 years (range 23-86 years) who had been using IC for a mean length of 8.5 years (range 9 months – 30 years). The frequency of IC ranged from once every second day to 10 catheterisations a day (mean 4/day). Reasons for IC included chronic urinary retention (n=20), neurological impairment (n=10) and other factors such as post-surgery or post-chemotherapy (n=9).

The majority of participants (n=34) were using a catheter once and discarding it after use. Most (n=30) were using hydrophilic coated catheters, of whom 10 used catheters with a compact design. Of the remaining five, four were currently reusing catheters designed for single-use on an on-going basis, and one had previously re-used catheters, switching to single-use 10 years ago because of convenience. The four participants who re-used catheters were all men who had been re-using for between 10 and 30 years. Their reasons for re-use were concerns about waste or cost.

Qualitative findings

For peer review only - http://bmjopen.bmj.com/site/about/guidelines.xhtml

The results focus on the perspectives of the participants regarding single-use and re-use of IC catheters; themes emerged about safety, burden and lifestyle. The four main themes were concerns regarding risk of UTI; cleaning, preparation and storage; social responsibility and practicalities and location. The themes and sub-themes are described below and shown in Figure 1. Those who had never re-used catheters were asked about their views but not their own experiences.

Overall, around half of the single-use participants indicated they would not want to re-use IC catheters or had major concerns about whether it would be an acceptable method. For the remaining participants, the potential acceptability of re-use was very much dependent on the safety and efficacy of the cleaning regimen and whether there was an increased risk of infection, as well as factors such as convenience and practicalities (Figure 1).

1. Concerns regarding risk of UTI

1.1 Safety: Fears about increasing risk of UTI

The question of UTI risk was the main concern raised by many single-users when asked about re-using catheters. This was the case for those who had suffered UTIs and others who had not and for men and women participants. They described their concerns about infection as their biggest worry and for some it was the only issue they had when considering re-use.

> *Infection [concerns me about re-using catheters] definitely. Especially as I've not had any infection, I think I'm doing something right, if you get me. (Participant 22, female, single-user)*

> *My honest opinion [about reusing] is I can understand why they're trying to do it, it's they're trying to save money. But in my opinion there's always a chance that that's going to go wrong and I'm the one who's gonna end up having a water infection or something like that. (Participant 13, male, single-user)*

Many perceived that using single-use catheters meant they had less chance of getting an infection and they felt that the single-use method was more 'clean' and 'sterile'.

> *[Single-use catheters have] less chance on infection, you can dispose of it, get rid of it. (Participant 40, male, single-user)*

However, the four participants who were re-using were not deterred by fears about infection.

For peer review only - http://bmjopen.bmj.com/site/about/guidelines.xhtml

1.2 Necessity of safe cleaning method

Related to the fears described above, some single-users described the importance of an effective catheter cleaning method as a pre-requisite for re-use.

> *[It is important] that you follow all the steps that you are supposed to. Because if you don't then it might not be sterile. Because I don't really know what it, what those effective ways would be. (Participant 16, female, single-user)*

2 Cleaning, preparation, and storage

2.1 Concerns about a complicated/time-consuming cleaning procedure

Many single-users thought that the cleaning procedure would be complicated and were concerned about the amount of work and time involved. Some did not want to be bothered with it and thought it could be inconvenient and potentially messy.

> *….but I mean if you had to go to all the bother of washing a catheter, I don't know how you'd do it to be quite honest, to get it really sterile. (Participant 29, female, single-user)*

Some single-users mentioned that they needed to know more about the cleaning method before they could comment on re-use. It appeared, however, that all four of the catheter re-users were conducting a very simple catheter cleaning procedure, typically using only tap water to rinse out used catheters.

> *Holding it under the tap [running it under tap water], and then I put it right back in the pouch, and then it's as clean as a whistle. […] (Participant 19, male, re-user)*

2.2 Concerns about storage and supply of catheters

The participants who would consider re-use of catheters thought it could save space in the home. However, some suggested that finding space to store extra equipment for cleaning catheters would be a problem. One participant, with a complex long-term health condition that required other equipment, was concerned about this due to the amount of equipment she already had.

> *My bathroom is already clogged full of all my bulky stuff, I already have my sort of like bag that hangs up for flushing my bowels out.[…] I*

For peer review only - http://bmjopen.bmj.com/site/about/guidelines.xhtml

think that I'd want it to not be too bulky. (Participant 11, female, single-user)

In terms of catheter supply, although some participants were very happy with their current supply arrangements, for others the prospect of a supply shortage was a drawback of single-use catheters. Some participants thought they would not need to worry about running out if they could re-use them.

Wouldn't have to keep running up the chemist [if I used reusable catheters]. You'd know you've always got one. You would know there's always one there. (Participant 7, female, single-user)

3 Social responsibility

3.1 Reducing costs to NHS

Many participants expressed their concerns or could see the issues around the financial cost of their catheters for the National Health Service (NHS) and some suggested that they would consider re-use to contribute to reducing such cost.

I think the GP's surgery pays for [my catheter] but I found out how much it was last week and I was shocked, really shocked actually. I feel guilty about that. (Participant 15, female, single-user)

Some described the potential cost-saving as the only benefit of re-use and noted that the benefit was not to them directly. Two participants questioned whether there would be any saving, with one considering that it may cost more due to the potential for increased infections.

3.2 Reducing waste and the impact on the environment

Several participants felt that re-using catheters would reduce waste and, for that reason, might be more environmentally friendly.

The good thing about re-using would be that it would cut down the amount of waste. If I go through [single-use catheters] every 6 weeks that is a mountain of catheters every year I get though. So I think that is the main benefit really. (Participant 38, female, single-user)

For peer review only - http://bmjopen.bmj.com/site/about/guidelines.xhtml

A participant who was re-using indicated that not disposing of the catheter after each use alleviated the worry about contributing to landfill waste. Another re-user described how reducing waste was the main motive to start reusing their catheters.

> *I hate wasting anything, and so that's all built into why I use the catheter more than once. (Participant 25, male, re-user)*

4 Practicalities and Location

4.1 Freedom and a normal life

Most single-users appreciated their current catheters as they were convenient, comfortable and easy to use, with some describing them as giving freedom and a normal life; IC had become part of life, a natural thing like cleaning teeth.

> *I love that [my catheter] is compact. They are just so convenient to use. No fuss. It doesn't impact on your normal daily lifestyle. They are great for travelling. That's what I love about my catheters. And it gives me a normal life; otherwise I would probably have to be at home more really. (Participant 38, female, single-user)*

The participants were often familiar with the design of their current single-use catheter and described how it suited them personally, although this may not directly relate to single-use or reusability.

When considering re-use, the participants discussed the locations where they would be more likely to re-use their catheters; they often compared the practicalities of re-use inside and outside the home as well as issues around travel.

4.2 Travel

The compact size of single-use catheters was appreciated by participants because they were easy to carry individually. However, for holidays, the need to take a large supply was a hindrance. In this situation, participants noted that re-use of catheters could be a more practical option.

One participant valued this aspect of reusing from his experience.

> *If I take [my catheters], I'll take 10 for the month or something. If I take the other one I've got to take 120. […] I did a trip around Australia once with my family and we were away 30 days or a month*

For peer review only - http://bmjopen.bmj.com/site/about/guidelines.xhtml

> *and the difference would have been huge. I pretty much would have needed a suitcase just for that. So that's always been a major issue. (Participant 37, male, re-user)*

However, several single-users were concerned about carrying the cleaning equipment on holiday.

4.3 Carrying used catheters

Many participants appreciated the portability and ease of disposal of the single-use catheters. Some single-users, therefore, wondered what they would have to do with the used catheters if they catheterised when they were out.

> *If you are going out for the day. You'd use your catheter in the morning; I don't want to take that one around with me. I don't know how I could. (Participant 41, male, single-user)*

However, one of the re-users emphasised the possible flexibility of reusing their catheter, as they could also be thrown away after a single use if needed.

> *You can always throw it away and you've only done the same as you've done with a non-re-usable one. So if you find yourself out and about and it's not as hygienic as you'd have liked; […] I will tend to use it once and throw it away under those circumstances. (Participant 37, male, 50, single-user)*

4.4 Discretion

The majority of single-users appreciated the compact packaging and discreetness of their current catheters. Many were concerned they would not have these advantages if they re-used catheters and the lack of discreetness would restrict them.

> *[Single-use catheters are] just quick really, it doesn't take me any longer than somebody else going into the toilet, not much longer at all so it's that easy. If you have to start cleaning it after using it….while you're out, it then gets a bit [much]. And it's obviously difficult to do it in a public toilet as well. (Participant 3, female, single-user)*

For peer review only - http://bmjopen.bmj.com/site/about/guidelines.xhtml

While some said that they would be happy to re-use at home, others expressed their concern, as they did not want visitors to see used catheters or the cleaning equipment.

4.5 Choice of catheters – mixed use

Among those who viewed re-use as potentially acceptable, most said they would value having both types of catheters to choose from depending on the situation.

> *Important stuff would be that when I'm doing it at home it's not generally a problem. If I know what I've got to do I will do it. It's knowing that when you are going away, you don't know where you are going to, […} is that equipment there? For me to be able to do it? (Participant 1, male, single-user)*

> *…..I wouldn't want to be forced to have just re-used catheters. But in most circumstances I would choose to use it accept for when I'm travelling in which case I would definitely want to be using the disposable ones. And I think generally speaking if you are away you don't want all the faff. (Participant 38, female, single-user)*

DISCUSSION

We believe this is the first study to explore IC users' perspectives on the advantages and disadvantages of single-use and re-use of catheters. The findings highlight the complexity of factors involved and show the variation of views amongst IC users; for some, re-use is not an acceptable option and for others it could be acceptable, but robust evidence is needed regarding safety, and the cleaning method must be shown to be effective and user-friendly.

Concerns regarding risk of UTI

For IC users to consider re-use they must first be satisfied that it is safe to do so; this was the crucial factor for many. In fact, around half of participants in this study said they would not consider re-use or had major concerns about it, mainly due to fears about infection. A guideline for the diagnosis, treatment and prevention of catheter associated UTI (IDSA) concluded that there have not been enough robust studies to provide evidence regarding risk of UTI when comparing re-use of catheters to single-use catheters.(5) This emphasises the need for a randomised controlled trial to properly examine this.

Cleaning, preparation and storage

The development of a cleaning method that is practical for IC users was an issue raised by participants. Concerns were voiced about the number of cleaning items requiring storage and the potential burden of doing the cleaning as well as the effectiveness of the cleaning method. Others have suggested that the cleaning method could be a burden when re-using catheters (14) which could in turn impair both independence and adherence;(15) Chick et al (2015) found that

For peer review only - http://bmjopen.bmj.com/site/about/guidelines.xhtml

some families in their study preferred the convenience of the one-step process for single-use catheters.(8) It is therefore important that the cleaning method is developed with IC users alongside rigorous laboratory testing.(16)

Social responsibility

Despite participants' concerns about re-use, they recognised some advantages. Many thought there could be cost savings for the NHS and a positive environmental impact and some were currently re-using, or would consider re-using, for these reasons. Similar findings were reported by Kelly in which 16 individuals were interviewed about their experiences and concerns about IC use. Financial burden to the health service and environmental impact were raised by 63% (10/16) and 38% (6/16) of the participants respectively; the authors suggested this reflects the greater awareness of environmental issues in society.(6) Although several of the participants in this study felt that re-use of catheters would be more environmentally friendly, this is a complex issue dependent on the method used for cleaning; there is a need for knowledge to be gained on environmental issues.

Four distinct modelling studies on cost effectiveness using different IC catheters and methods have been published. Both Neovius(17) and Bermingham(18) compared re-use with single-use catheters. In a conference abstract, Neovius concluded that re-use of PVC catheters was not cost effective compared to single-use coated catheters,(17) whereas Bermingham indicated that re-use could be more cost effective than single-use catheters but that additional evidence was required before any practice changes could be recommended.(18) Clark and colleagues(19) concluded that single-use coated rather than single-use uncoated catheters were more cost effective when outcomes were calculated over a patient's lifetime. Neovius and Lundquist(20) indicated that patients would be willing to pay out of pocket for a hypothetical catheter that would reduce/prevent UTIs. The probabilistic decision model of these studies regarding risk of UTI limits the interpretation of the results and indicates, as Bermingham stated, that further robust evidence is required.

Practicalities and location

Although many of the participants would consider catheter re-use, willingness to do so varied between individuals based on lifestyle and circumstances. The need for discretion was of great concern and was closely linked to location; some participants would only consider re-using catheters when they were at home. The literature also confirms that location, and specifically whether at home or away from home, has a bearing on how people feel about re-use.(8, 9) This highlights the need for a flexible approach to IC methods, which may be achieved by giving IC users the option to use both catheter types to suit their lifestyle and preferences.

Many of the perceived disadvantages of re-use are off-set by advantages of single-use and vice versa (see Figure 1). For example, some IC users were concerned about running out of their single-use catheters, and the ability to re-use their catheter would mean they always have a usable catheter. Others emphasised the benefit of using single-use catheters when in public

For peer review only - http://bmjopen.bmj.com/site/about/guidelines.xhtml

places and the difficulties of re-use when away from home reinforcing the potential benefit of mixed use. Furthermore, if re-use of catheters was more commonplace, efforts could be made to design and manufacture discreet, re-usable versions in the same way as for single-use.

Limitations

As the primary aim of the study was to explore whether reusing catheters would be acceptable to IC users in preparation for a randomised controlled trial, and this was stated in the participant information sheet, we may have recruited primarily individuals who were interested in issues around re-use. Therefore, findings may not be representative of all UK IC users. Furthermore, the perspectives of single-use IC users about re-using catheters were hypothetical and not based on their actual experiences. The inclusion of questions about advantages and disadvantages of both methods may have influenced participants responses and the researcher's presence during the data collection might have elicited socially desirable responses; however, the researcher presented as an impartial observer during the data collection and every effort was made to keep the interviews balanced.

Implications for future research/clinicians/policy makers

This study has shown that some IC users would find re-use of catheters acceptable, but there is a need to develop a rigorous cleaning method for IC catheters which is effective and user-friendly. If shown to be safe, then efforts could be made to develop user-friendly catheter designs, e.g. compact designs, with an efficient cleaning method.

CONCLUSION

This study revealed that if shown to be safe with a practical cleaning method, some participants would find re-use of catheters for some of the time an acceptable option. The choice to use a mixture of single-use and re-use of catheters for different activities (at home, work or holiday) could optimise the advantages and disadvantages of both methods. With environmental issues and cost high on the public agenda, some IC users would advocate that if re-use is shown to be safe and acceptable then it could be made available as an option. The safety and acceptability of such an approach would require testing in a clinical trial.

Acknowledgements

We would like to thank all the interviewees who agreed to take part and share their experiences.

Contributors

MF was the chief investigator and was primarily responsible for the original grant application. MF, JP, MM, SC and MA were involved in designing the study and methods. JP led the overall study team. SC collected the majority of the data. BC and MA contributed to the data collection. IO led the analysis and IO and MA were involved in interpretation of the qualitative data and

For peer review only - http://bmjopen.bmj.com/site/about/guidelines.xhtml

wrote draft manuscripts. IO, JP, MA, KNM, MM and MF wrote drafts and/or critically revised the manuscripts.

Patient consent

Detail has been removed from this case description/these case descriptions to ensure anonymity. The participants provided consent to publish anonymous quotes.

Competing interests

The authors of the papers were funded by the NIHR under its Programme Grants for Applied Research (PGfAR) (Grant Reference Number RP-PG-0610-10078).

Data sharing statement

No additional data is available.

REFERENCE LIST

1. McConville A. Patients' experiences of clean intermittent catheterisation. *NTplus* 2002;98(4):55-56.
2. Shaw C, Logan K, Webber I, et al. Effect of clean intermittent self-catheterization on quality of life: a qualitative study. *J Adv Nurs* 2008;61(6):641-50.
3. Kessler TM, Ryu G, Burkhard FC. Clean intermittent self-catheterization: a burden for the patient? *Neurourol Urodyn* 2009;28:18-21.
4. Bolinger R, Engberg S. Barriers, complications, adherence, and self-reported quality of life for people using clean intermittent catheterization. *J Wound Ostomy Continence Nurs* 2013;40(1):83-89.
5. Hooton TM, Bradley SF, Cardenas DD, et al. Diagnosis, prevention, and treatment of catheter-associated urinary tract Infection in adults: 2009 international clinical practice guidelines from the Infectious Diseases Society of America. *Clin Infect Dis* 2010;50:625-63.
6. Kelly L, Spencer S, Barrett G. Using intermittent self-catheters: experiences of people with neurological damage to their spinal cord. *Disabil Rehabil* 2014;36(3):220-226.
7. Wilde MH, Brasch J, Zhang Y. A qualitative descriptive study of self-management issues in people with long-term intermittent urinary catheters. *J Adv Nurs* 2011;67(6):1254-63.
8. Chick HE, Hunter KF, Moore KN. Parent and child experiences using a hydrophilic or reused PVC catheter for intermittent catheterisation. *J Clin Nurs* 2012;22:513-520.
9. Kiddoo D, Sawatzky B, Bascu C-D, et al. Randomized crossover trial of single use hydrophilic coated versus multiple use polyvinylchloride catheters for intermittent catheterization to determine incidence of urinary infection. *J Urol* 2015;194:174-179.
10. Fader M. Development and clinical trial of a mixed (multi/single-use) catheter management package for users of intermittent catheterisation. *National Institute for Health Research (NIHR) under its Programme Grants for Applied Research (PGfAR)*. Programme (Grant Reference Number RP-PG-0610-10078), 2013
11. McClurg D, Coyle J, Long A, et al. A two phased study on health care professionals' perceptions of single or multi-use of intermittent catheters. *Int J Nurs Stud* 2017;72:83-90.

For peer review only - http://bmjopen.bmj.com/site/about/guidelines.xhtml

12. Okamoto I, Prieto J, Avery M, et al. Intermittent catheter users' symptom identification, description and management of urinary tract infection: a qualitative study. *BMJ Open* 2017;7: e016453. doi:10.1136/bmjopen-2017-016453.
13. Braun V, Clarke V. Using thematic analysis in psychology. *Qual research psychol* 2006;3(2):77-101.
14. Woodward S. Improving quality of life for men using intermittent self-catheterisation. *British Journal of Neuroscience Nursing.* 2013;9(3):114-119.
15. Dean G. Are Single Use Catheters Worth the Expense? *J Urol* 2015;194(1):12-3.
16. Wilks S, Morris N, Delgado D, et al. Development of an effective and acceptable cleaning method to allow safe re-use of plain, uncoated catheters for intermittent catheterization. *Neurourol Urodyn* (Abstract 566) 2016;4:449-450.
17. Neovius K, Hakansson M, Lundqvist T. Cost consequences of single-use and re-use of urinary catheters among patients performing daily intermittent catheterisation (Abstract PMD43). *Value in Health* 2014;18:A351.
18. Bermingham SL, Hodgkinson S, Wright S, et al. Intermittent self catheterisation with hydrophilic, gel reservoir, and non-coated catheters: a systematic review and cost effectiveness analysis. *BMJ* 2013; 346:e8639
19. Clark JF, Mealing SJ, Scott DA, et al. A cost-effectiveness analysis of long-term intermittent catheterisation with hydrophilic and uncoated catheters. *Spinal Cord* 2016;54(1):73-77.
20. Neovius KE, Lundqvist T. CIC users' preference for catheters reducing the UTI frequency (Abstract PUK14). *Value in Health* 2014;17:A468.

Figure Legend

Figure 1: Themes, sub-themes and coding from analysis of qualitative interviews

For peer review only - http://bmjopen.bmj.com/site/about/guidelines.xhtml

Main theme	Sub-theme	Codes			
		Re-use		Single-use	
		Advantages	Disadvantages	Advantages	Disadvantages
1. Concerns regarding risk of UTI	1.1 Safety: fears about increasing risk of UTI		Concern about hygiene and perceived link to UTI	Considered less likely to cause infection<br>Sterile	
	1.2 Necessity of safe cleaning		Cleaning and preparation		
2. Cleaning, preparation and storage	2.1 Concerns about a complicated, time-consuming cleaning procedure		Cleaning and preparation		
	2.2 Concerns about storage/supply of catheters	Always having one<br>No storage problem	Storing equipment<br>Concern use-by-date could expire	Good delivery system/good relationship with supplier	Shortage of supply/not good delivery service
3. Social responsibility	3.1 Reducing costs to NHS	Cost			Cost
	3.2 Reducing waste/impact on environment	Environment			Environment and waste
4. Practicalities and location	4.1 Freedom and a normal life		Limiting lifestyle	Instantly usable or easy to use and to dispose<br>No pain and easy to use<br>Gives freedom/normal life<br>More independence	
	4.2 Travel	Less to carry when travelling	Taking cleaning equipment on holiday		Not easy to carry on holiday
	4.3 Carrying used catheters		Carrying used catheters		
	4.4 Discretion		Not discreet	Easy to carry and discreteness<br>Ready to use<br>Easy to use	Don't like to dispose used catheters in public toilets<br>Not easy to carry around
	4.5 Choice of catheters – mixed use	Emerged from interviews			

Figure 1: Themes, sub-themes and coding from analysis of qualitative interviews

108x60mm (300 x 300 DPI)

review only

MultICath Module 2 Schedule for iteration 3

Background questions

1. Experience of intermittent catheterisation:

How long have you been using intermittent catheterisation?

Do you self-catheterise? Or does somebody help you?

How many times per day do you catheterise?

What catheter do you use?

Do you catheterise:

At home only?

When you are out?

If yes, when you are out what kind of catheter do you use, is it the same one as for home use?

Beliefs and practices re IC routine

2. Beliefs and concerns about IC rituals, catheters, infection and sterility

What steps do you take before you do IC?

What is the reason you take these steps? (Probe without leading for whether or not they value sterility)

Is your IC routine clean or sterile?

What is sterile to you? (prompt – how is it different to clean?) What do you mean by clean?

Experience of urine infection

3. During your time using intermittent catheterisation have you ever experienced a urine infection?

If yes, how often would you say you have a urine infection?

Can you recall the last time you had a UTI? What happened?

4. What it feels like to have a urine infection:

Can you describe how you feel when you think you may be developing a urine infection?

Does anything happen beforehand when you think one is coming on?

For peer review only - http://bmjopen.bmj.com/site/about/guidelines.xhtml

How do you think your infection came about? Prompt (Where do you think the germs come from when you get a UTI?)

What do you do to prevent an infection?

What advice do you get/have you had from your health care professional to prevent infections?

Complete the table – UTI signs and symptoms: Where patients name a symptom or sign check with them the terminology and that the patient is happy that it sits within a category on the table. We want to understand the terminology that patients use to describe UTI symptoms.

Looking at the symptoms listed here, are these the right things that describe a urine infection from your experience? Is anything missing?

5. Seeking help:

When you feel you may have a urine infection, what do you do about it?

Prompt: antibiotics

At what point do you seek help from others?

Who do you seek help from? And what do you do?

Cleaning questions

6. Methods of cleaning:

Have you ever re-used your catheter?

If Yes, under what circumstances? Did you clean it after its first use? How did you clean it? How did you store it between uses?

What is/would be acceptable to you?

If No, Have you ever thought about re-using your catheters?

If you were offered a method of cleaning and re-using your catheters instead of using a new one each time, what kinds of things would be important to you? [Prompts: How to clean; Where to clean; Where to store between uses]

What kind of cleaning regime is acceptable to you? [Prompts: what cleaning methods and products, what about storage storage/travelling, and lubrication?]

What would worry you about re-using your catheter? [Prompts: cleaning in a public loo, being at work, storing and transporting]

What would be good about re-using?

For peer review only - http://bmjopen.bmj.com/site/about/guidelines.xhtml

Discrete choice experiment questions

[I would now like to discuss with you the benefits and drawbacks of using disposable and reusable

Catheters]

7. What is good about your current catheter?

What do you think about single-use catheters?

What are the benefits of using a disposable or single-use catheter?

What are the drawbacks?

[in the following situations]

- At home?
- At work?
- On holiday?
- In a public place?
- Going out?
- Other?

What do you think about the idea of a re-usable catheter?

- What do you see as a benefit of re-using your catheter?
- What do you see as a drawback in re-using your catheter?

[in the following situations]

- At home?
- At work?
- On holiday?
- In a public place?
- Going out?
- Other?

- What would encourage you to use it?
- What would discourage you from using it?

Preamble: We know from previous interviews/speaking to other patients, that avoidance of infection is what matters most to everyone. We also know that other things are highly important such as:

- a comfortable insertion,
- having a catheter that is easy to use and handle,
- that comes in packaging which is easy to open,
- that your catheter is discrete,
- that you can trust your catheter/ trust in the product

As we already know all of this, we now want to explore other things around single-use catheters and re-usable catheters.

For peer review only - http://bmjopen.bmj.com/site/about/guidelines.xhtml

Together let's go through each item in this list and tell me what you think about each one? Your comments are very valuable to us.

Those relating to disposable catheters:

1. That I don't have the bother of cleaning the catheter
2. That I don't need to have special equipment for cleaning the catheter
3. That I don't have to carry used/soiled catheters around with me in my bag

Those relating to re-using catheters:

4. That I have less catheters to carry with me on holiday
5. That my catheters don't cost the NHS more than they need to/too much
6. That I don't have to store lots of catheters at home
7. That I don't have to worry that I will run out of catheters
8. That I don't have to get rid of lots of rubbish when I am out
9. That my catheters are not bad for the environment.

For peer review only

For peer review only - http://bmjopen.bmj.com/site/about/guidelines.xhtml

COREQ (COnsolidated criteria for REporting Qualitative research) Checklist

A checklist of items that should be included in reports of qualitative research. You must report the page number in your manuscript where you consider each of the items listed in this checklist. If you have not included this information, either revise your manuscript accordingly before submitting or note N/A.

Topic	Item No.	Guide Questions/Description	Reported on Page No.
**Domain 1: Research team and reflexivity**			
Personal characteristics			
Interviewer/facilitator	1	Which author/s conducted the interview or focus group?	
Credentials	2	What were the researcher's credentials? E.g. PhD, MD	
Occupation	3	What was their occupation at the time of the study?	
Gender	4	Was the researcher male or female?	
Experience and training	5	What experience or training did the researcher have?	
Relationship with participants			
Relationship established	6	Was a relationship established prior to study commencement?	
Participant knowledge of the interviewer	7	What did the participants know about the researcher? e.g. personal goals, reasons for doing the research	
Interviewer characteristics	8	What characteristics were reported about the inter viewer/facilitator? e.g. Bias, assumptions, reasons and interests in the research topic	
**Domain 2: Study design**			
Theoretical framework			
Methodological orientation and Theory	9	What methodological orientation was stated to underpin the study? e.g. grounded theory, discourse analysis, ethnography, phenomenology, content analysis	
Participant selection			
Sampling	10	How were participants selected? e.g. purposive, convenience, consecutive, snowball	
Method of approach	11	How were participants approached? e.g. face-to-face, telephone, mail, email	
Sample size	12	How many participants were in the study?	
Non-participation	13	How many people refused to participate or dropped out? Reasons?	
Setting			
Setting of data collection	14	Where was the data collected? e.g. home, clinic, workplace	
Presence of non-participants	15	Was anyone else present besides the participants and researchers?	
Description of sample	16	What are the important characteristics of the sample? e.g. demographic data, date	
Data collection			
Interview guide	17	Were questions, prompts, guides provided by the authors? Was it pilot tested?	
Repeat interviews	18	Were repeat inter views carried out? If yes, how many?	
Audio/visual recording	19	Did the research use audio or visual recording to collect the data?	
Field notes	20	Were field notes made during and/or after the inter view or focus group?	
Duration	21	What was the duration of the inter views or focus group?	
Data saturation	22	Was data saturation discussed?	
Transcripts returned	23	Were transcripts returned to participants for comment and/or	

For peer review only

Topic	Item No.	Guide Questions/Description	Reported on Page No.
		correction?	
**Domain 3: analysis and findings**			
Data analysis			
Number of data coders	24	How many data coders coded the data?	
Description of the coding tree	25	Did authors provide a description of the coding tree?	
Derivation of themes	26	Were themes identified in advance or derived from the data?	
Software	27	What software, if applicable, was used to manage the data?	
Participant checking	28	Did participants provide feedback on the findings?	
Reporting			
Quotations presented	29	Were participant quotations presented to illustrate the themes/findings? Was each quotation identified? e.g. participant number	
Data and findings consistent	30	Was there consistency between the data presented and the findings?	
Clarity of major themes	31	Were major themes clearly presented in the findings?	
Clarity of minor themes	32	Is there a description of diverse cases or discussion of minor themes?	

Developed from: Tong A, Sainsbury P, Craig J. Consolidated criteria for reporting qualitative research (COREQ): a 32-item checklist for interviews and focus groups. *International Journal for Quality in Health Care*. 2007. Volume 19, Number 6: pp. 349 – 357

Once you have completed this checklist, please save a copy and upload it as part of your submission. DO NOT include this checklist as part of the main manuscript document. It must be uploaded as a separate file.

For peer review only
